# Traditional Chinese medicine-derived natural photosensitizers for photodynamic therapy against pathogenic infections

**DOI:** 10.3389/fcimb.2026.1932770

**Published:** 2026-10-23

**Authors:** Jinju Huang, Siu Kan Law, Albert Wing Nang Leung, Chuanshan Xu

**Affiliations:** 1 Intensive Care Unit Ward 1, The Affiliated Panyu Central Hospital, Guangzhou Medical University, Guangzhou, China; 2 Independent Researcher, Hong Kong, China; 3 School of Graduate Studies, Lingnan University, Hong Kong, China; 4 Guangzhou Municipal and Guangdong Provincial Key Laboratory of Molecular Target & Clinical Pharmacology, The NMPA and State Key Laboratory of Respiratory Disease, School of Pharmaceutical Sciences, Guangzhou Medical University, Guangzhou, China

**Keywords:** clinical translation, nanotechnology, pathogenic infection, photodynamic therapy, TCM–derived photosensitizer

## Abstract

Photodynamic therapy (PDT) has emerged as a promising non-antibiotic strategy for combating pathogenic infections through the generation of reactive oxygen species (ROS) from a light-activated photosensitizer (PS), which interrupts microbial structures and functions. Natural products such as coumarin, curcumin, riboflavin, pheophorbide a, and hypericin have long been studied as PSs that have the functions of intrinsic antimicrobial, antiviral, antifungal, and antiparasitic activities. These metabolites are broadly distributed across multiple ethnomedical traditions worldwide, with a focus on those explicitly documented in the Chinese Pharmacopoeia and widely applied in Traditional Chinese Medicine (TCM), while acknowledging their broader global distribution. Natural PSs have some limitations for PDT efficacy. Recent advances in nanotechnology and drug delivery systems have enabled the development of TCM-derived advanced PSs with improved solubility, bioavailability, and targeted accumulation at infection sites. This narrative review was conducted through a comprehensive search of multiple electronic databases, including PubMed, ScienceDirect, Scopus, Web of Science, and the China National Knowledge Infrastructure (CNKI). The search encompassed literature published over the past three decades, without restrictions on language. Studies were considered eligible if they contained the keywords “Photodynamic Therapy”, “Pathogenic infection”, “Coumarin”, “Curcumin”, “Riboflavin”, “Pheophorbide a”, and “Hypericin”. Duplicate records were excluded during screening. All relevant publications were collected, critically appraised, and synthesized to provide mechanistic insights, preclinical evidence, and translational perspectives on the application of TCM–derived PSs in photodynamic therapy against bacterial biofilms, viral pathogens, and fungal infections. The synergistic benefits include overcoming antimicrobial resistance, enhancing host immune responses, and reducing reliance on conventional antibiotics. Challenges remain in optimizing pharmacokinetics, minimizing off-target toxicity, and advancing clinical validation or translation. Thus, TCM-derived advanced PS represent a novel and integrative approach to fight against pathogenic infection, bridging ethnopharmacology with modern photomedicine, and hold significant potential or advantages for clinical application in the future.

## Graphical Abstract

**Figure:**
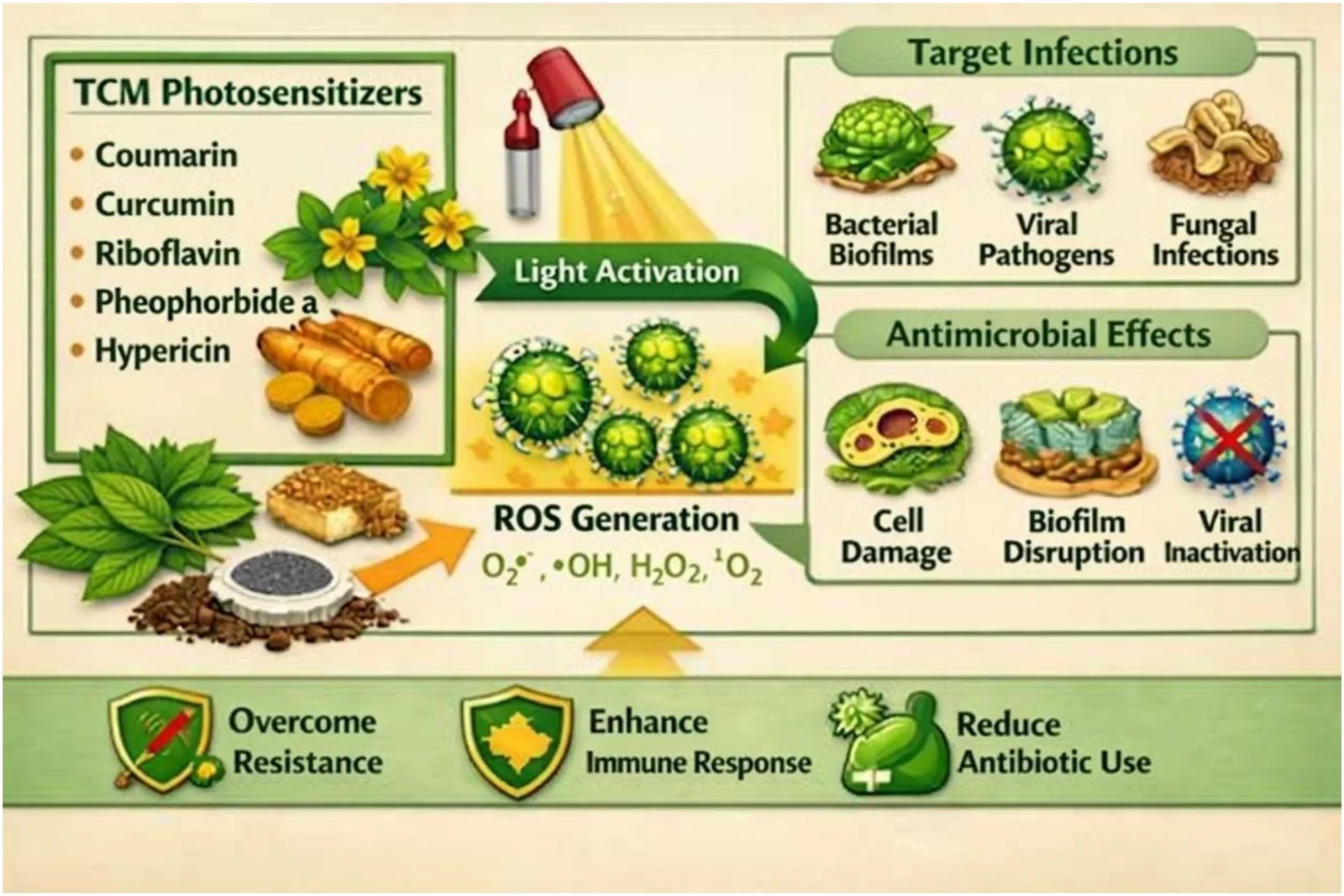

## Introduction

Pathogenic infections are illnesses caused by microorganisms, including viruses, bacteria, fungi, and parasites. They invade host tissues, evade immune defenses, and disrupt normal cellular processes. Virulence, or the severity of infection, is determined by the pathogen’s ability to penetrate tissues, circumvent immune responses, and secrete toxins to inflict cellular damage (Balloux and van Dorp, 2017).

The treatment of pathogenic infections typically involves a combination of conventional antimicrobials, which requires matching the specific microbe to the appropriate antimicrobial drug class (Belay et al., 2025), such as antibiotics, antifungals, antivirals, and antiparasitics. Antibiotics are prescribed to combat bacterial infections, including strep throat and urinary tract infections. They function by either impairing the synthesis of bacterial cell walls or blocking protein production, ultimately preventing the bacteria from multiplying and causing further harm (Grennan et al., 2020). Antivirals are prescribed to manage viral infections, like influenza and HIV. They treat the infection through reducing viral load, alleviating symptoms, and shortening the duration of illness (Drexler and Institute of Medicine (US), 2010). Antifungal agents are used to eradicate fungal and yeast infections, such as ringworm and cryptococcal meningitis, which target sterols within the fungal cell membrane, compromising its integrity and leading to the destruction of the pathogen (Li et al., 2025). Antiparasitic agents treat infections caused by parasites, e.g., malaria and giardiasis. They interfere with metabolic pathways or disrupt the parasite’s ability to survive and reproduce within the host (Rosenblatt, 1999).

However, antibiotics, antivirals, antifungals, and antiparasitics remain the cornerstone for treating pathogenic infections. The limitations include resistance, toxicity, narrow spectrum, and incomplete eradication (Abbas et al., 2024; Doudna, 2023), which highlight the urgent need for novel therapy, such as antimicrobial photodynamic therapy (aPDT). Compared with the conventional treatments of pathogenic infections, aPDT has distinct advantages involving eradicating drug-resistant strains, as the multidrug-resistant (MDR) superbugs remain susceptible to reactive oxygen species (ROS), restoring antimicrobial efficacy in cases where conventional antibiotics are ineffective (Demidova and Hamblin, 2004). Meanwhile, aPDT effectively penetrates and disrupts dense bacterial biofilms for protective matrices that typically shield pathogens from conventional antibiotic treatments (Tanu et al., 2025). Besides, aPDT has broad-spectrum activity, which demonstrates strong efficacy against diverse pathogens, including Gram-positive and Gram-negative bacteria, fungi, viruses, and protozoa (Gilaberte et al., 2021). More importantly, aPDT is highly targeted, since the light activation is confined to the infection site, surrounding healthy tissues are spared, and systemic toxicity is minimized (Amendola et al., 2025).

In light of these limitations, aPDT emerges as a promising non-antibiotic approach. TCM offers a unique reservoir of natural photosensitizers that not only embody ethnopharmacological principles but also demonstrate intrinsic antimicrobial, antiviral, antifungal, and antiparasitic activities, thereby bridging traditional knowledge with therapeutic strategies for pathogenic infections. Beyond their natural forms, these compounds have been engineered into advanced PSs through nanotechnology and formulation strategies, thereby enhancing their solubility, bioavailability, and targeted accumulation, and ultimately strengthening their clinical potential.

The term ‘TCM-derived photosensitizers’ refers specifically to natural products documented in the Chinese pharmacopoeia or widely recognized in TCM practice. These compounds are usually used in other ethnomedical traditions worldwide based on their TCM context and therapeutic relevance. They have been applied in Chinese medicine for functions described as “invigorating blood circulation”, “clearing heat”, or “resolving toxicity”. The classification highlights their ethnopharmacological context in TCM while acknowledging their broader distribution and usage across other traditional systems.

Unlike previous studies that primarily catalogued antimicrobial agents and their mechanisms by focusing narrowly on resistance trends or single drug classes, this study integrates evidence across antibiotics, antivirals, antifungals, and antiparasitics. The aPDT has broad-spectrum efficacy, biofilm disruption, and targeted action, providing a more comprehensive synthesis of the advances in therapeutic perspectives. Therefore, this review aims to explore the aPDT as a promising alternative strategy through the use of TCM as PSs, including coumarin, curcumin, riboflavin, pheophorbide a (PPa), and hypericin. To examine its mechanisms, including the generation of ROS, disruption of biofilms, broad-spectrum efficacy, and targeted action. The work of aPDT addresses critical challenges such as drug resistance and systemic toxicity, as well as current research processes. The discussion integrates evidence and emerging applications to provide a comprehensive perspective on the role of aPDT in advancing infection control and shaping future therapeutic strategies against pathogenic microorganisms (**Figure 1**).

**Figure 1:**
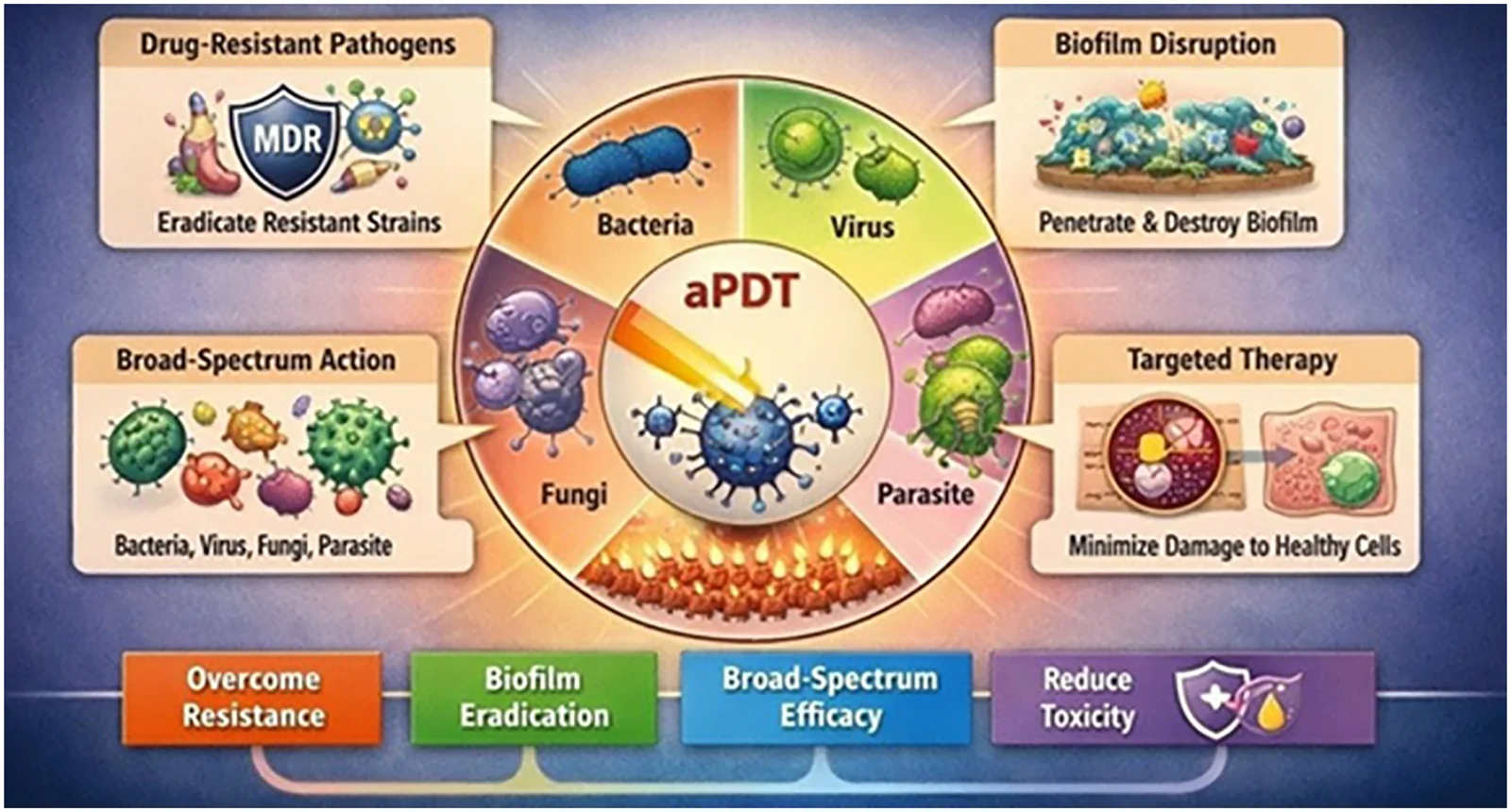
Over workflow of PDT against pathogenic infections.

## Search strategy

This narrative review was conducted using search strings across major databases, including PubMed, Scopus, Web of Science, and the China National Knowledge Infrastructure (CNKI). The search covered publications from 1995 to 2026, guided by keywords such as “Coumarin” OR “Curcumin” OR “Riboflavin”, “Pheophorbide a” OR “Hypericin” AND “Photodynamic Therapy” OR “Pathogenic infection”. The study findings were presented and summarized without language restrictions (**Supplementary File 1**). Regarding the search strategy, it specifically targeted studies on TCM-derived PSs, photophysical features, chemical structure, and PDT mechanism.

Records were screened in two stages: (1) titles and abstracts were reviewed to exclude irrelevant studies, and (2) full texts were assessed for eligibility. Inclusion criteria comprised English-language studies that investigated TCM-derived PSs in PDT against pathogenic infections. Exclusion criteria included conference abstracts without full papers and studies lacking methodological detail. The selection workflow identified 4120 records: 1245 from PubMed, 1030 from Scopus, 980 from Web of Science, and 865 from CNKI. 720 duplicates were removed. 3400 records remained for screening. Title and abstract screening excluded 2600 records as irrelevant. The remaining 800 full-text articles were assessed for eligibility, of which 748 were excluded (non-PDT focus, insufficient methodological detail, or conference abstracts only). Lastly, 52 studies were selected to fulfill the inclusion criteria and were synthesized in this narrative review (**Supplementary File 1**, **Supplementary Table S1**).

## TCM-derived PSs

Coumarin, curcumin, riboflavin, PPa, and hypericin are natural products derived from herbal or traditional Chinese medicinal plants. They combine TCM theory and intrinsic pharmacological activities to have antimicrobial, antiviral, antifungal, and antiparasitic effects.

Coumarin exhibits strong antimicrobial and antiviral effects, including inhibition of *Staphylococcus aureus* (*S. aureus*) and HIV, while curcumin shows efficacy against bacteria, resistant *Candida* strains, and parasites in aquaculture (Mishra et al., 2020; Hussain et al., 2022; Hajifathali et al., 2023). Riboflavin enhances host immunity and inhibits *Schistosoma mansoni* proteases (Farah et al., 2022; Cogo et al., 2024). Chlorophyll derivatives such as PPa disrupt bacterial biofilms and demonstrate virucidal or antiparasitic activity (Tayler et al., 2019; Jimenez-Aleman et al., 2021). Hypericin reduces MRSA virulence, exhibits antifungal activity against *Candida*, and induces necrosis-like death in *Leishmania* (Wang et al., 2019; Sytar et al., 2016; Singh et al., 2015) (**Table 1**). They are documented in the Chinese pharmacopoeia. These findings highlight the integrative potential of TCM-based photosensitizers in photodynamic therapy, emphasizing their photophysical features, chemical structures, mechanisms, and therapeutic effects against pathogenic infections. In addition to their intrinsic pharmacological activities, these natural products have been reformulated into advanced PSs using nanocarriers, liposomes, and polymer conjugates, which significantly improve their therapeutic performance in PDT.

**Table 1:** Pharmacological activities of natural product PSs derived from green or traditional Chinese medicinal plants.

Natural product	Source	TCM theory
Coumarin	Dang Gui (*Angelica sinensis*) (Choi et al., 2022) Qianhu (*Peucedanum praeruptorum*) (Lin et al., 2024)	Invigorate blood circulation, dispel blood stasis, clear heat, and reduce inflammation (Mustafa, 2025)
Pharmacological activities & consequences		
a. Antimicrobial Demonstrate notable antimicrobial activity, particularly against *S. aureus*, and structure–activity relationship studies indicate that halogenation, bulky aromatic substituents, nitro and hydroxyl groups enhance the interaction of coumarin rings with amino acid residues (Peter and Sibali, 2025) b. Antiviral Has the inhibitory role against infection of various viruses such as HIV, Influenza, Enterovirus 71 (EV71) and coxsackievirus A16 (CVA16), which involve the mechanisms in either inhibition of proteins essential for viral entry, replication and infection or regulation of cellular pathways such as mammalian target of rapamycin (Akt-Mtor), nuclear factor kappa-light-chain-enhancer of activated B cells (NF-kB), and anti-oxidative pathway including Nuclear factor erythroid 2-related factor 2 (NrF-2) (Mishra et al., 2020) c. Antifungal Induce *Candida albicans* apoptosis associated with mitochondrial Ca^2+^ influx for low toxicity and effectiveness in prolonging the survival of *C. albicans*-infected mice (Jia et al., 2019) d. Antiparasitic Interfere with parasite metabolism, disrupt mitochondrial function, inhibit key enzymes, and modulate host immune responses *in vitro* and *in vivo* efficacy with good selectivity toward the parasite (Tariq et al., 2025)		

Natural product	Source	TCM theory
Curcumin	*Curcuma longa* L. (turmeric) rhizome (Sharifi-Rad et al., 2020)	Promote the movement of Qi, invigorate blood circulation, and resolve blockages to relieve pain (Wasim et al., 2025)
Pharmacological activities & consequences		
a. Antimicrobial Effective against gram-positive bacteria, *S. aureus*, and gram-negative strains, *Escherichia coli*, which induce oxidative stress and suppress bacterial virulence factors (Hussain et al., 2022) b. Antiviral Interfere with the replication and cell-attachment processes of viruses, like Zika, influenza, and herpes simplex (Hussain et al., 2022) c. Antifungal Inhibit the proliferation of nystatin-resistant C. albicans strains (Hajifathali et al., 2023) d. Antiparasitic Safe and effective as a therapeutic for controlling ichthyophthiriasis in aquaculture, and completely terminate the reproduction of tomonts (Liu et al., 2017)		

Natural product	Source	TCM theory
Riboflavin	Goji Berry (*Lycium chinense*) (Tuan et al., 2014)	Replenish internal energy (qi) and invigorate the spleen (da Silva-Araújo et al., 2025)
Pharmacological activities & consequences		
a. Antimicrobial Modulate host immune responses, which may increase pathogen clearance from host cells and increase host defense against microbial infections (Farah et al., 2022) b. Antiviral RNA-targeted therapeutic agent against SARS-CoV-2 during viral inoculation, which significantly reduce viral replication (Jeong et al., 2025) c. Antifungal Reduce *Cryptococcus neoformans*, modulate lung and brain damage, and decrease the levels of plasma interferon (IFN)-γ, tumor necrosis factor (TNF)-α, and interleukin (IL)-4 in the early stage of infection (Huang et al., 2025) d. Antiparasitic Inhibit the activity of cathepsin B1 cysteine protease (SmCB1) of *Schistosoma mansoni* at low micromolar values and kill the parasite *in vitro* (Cogo et al., 2024)		

Natural product	Source	TCM theory
PPa	Noni (Morinda citrifolia) (Ratnoglik et al., 2014)	Manifestation of blood stasis, damp-heat, and toxic accumulation (Liu et al., 2015)
Pharmacological activities & consequences		
a. Antimicrobial Demonstrate the ability to act against pathogens like *Escherichia coli* and methicillin-resistant *S. aureus* (MRSA), which destroy their cell membranes and effectively penetrate the protective extracellular matrix of bacterial biofilms (Namulinda et al., 2024) b. Antiviral Exhibit photoactivable, broad-spectrum virucidal activity, interfere with virus-cell fusion, and reduce the replication of enveloped RNA viruses, including SARS-CoV-2 (Jimenez-Aleman et al., 2021) c. Antifungal Induce ROS-mediated oxidative stress and cell death, destroy the cell membrane and hyphal morphology, as well as ultimately inhibit mycelial growth of *C. oleifera* (Shi et al., 2024) d. Antiparasitic Demonstrate activity against parasites, such as *Trypanosoma cruzi*, the causative agent of Chagas disease, and *Leishmania amazonensis* (Tayler et al., 2019)		

Natural product	Source	TCM theory
Hypericin	St. John’s Wort (*Hypericum perforatum*) (Danova et al., 2022)	Clear heat and resolve toxicity, balance internal harmony (Yang et al., 2024)
Pharmacological activities & consequences		
a. Antimicrobial Reduce *sarA* expression and subsequently decrease *mecA* and virulence-related regulators (*e*.*g*., *agr RNAIII*) and genes (*e*.*g*., *fnbA* and *hla*) expression in the studied MRSA strain (Wang et al., 2019) b. Antiviral Reduce viral titers, reveal stable interactions with conserve residues in RdRp and 3CLpro for a low risk of resistance (Souza et al., 2025) c. Antifungal Exhibit antifungal activity against pathogenic fungi, including *Candida* species, with the capacity to inhibit fungal growth and reduce viability (Sytar et al., 2016) d. Antiparasitic Possess antileishmanial activity, and the mode of death show necrosis-like features, which decrease trypanothione levels and increase glutathione levels with elevate ROS levels (Singh et al., 2015)		

Coumarin, curcumin, riboflavin, PPa, and hypericin demonstrate broad antimicrobial, antiviral, antifungal, and antiparasitic activities, with mechanisms ranging from ROS generation to immune modulation, which highlight how natural PSs bridge ethnopharmacology with modern PDT applications.

## Photophysical feature

Coumarin, curcumin, riboflavin, PPa, and hypericin are TCM-derived PSs in PDT, which exhibit distinct photophysical characteristics to achieve their therapeutic effects. These features include absorption (λ_abs_), excitation (λ_ex_), emission (λ_em_), singlet oxygen quantum yield (Φ_Δ_), and dual functionality to determine how effectively each compound functions as a PS. Absorption and excitation spectra define the optimal light source for activation, while emission spectra provide diagnostic fluorescence signals. ROS generation and quantum yield reflect the efficiency of oxidative stress induction against pathogens. Dual functionality is the combination of light-activated ROS production with intrinsic pharmacological activities, such as antimicrobial, antiviral, antifungal, and antiparasitic effects, which are characteristic of many natural product PSs derived from traditional Chinese medicinal plants.

The photophysical features of TCM-derived PSs have a wide range of the spectrum from ultraviolet to visible light (**Table 2**). Coumarin operates at the shortest absorption wavelengths from ~320–380 nm with low singlet oxygen production. Curcumin and Riboflavin utilize blue-light excitation from 420–445 nm, generating moderate-to-high yields up to 0.60 and 0.54, respectively. PPa and Hypericin operate efficiently in the deeper red therapeutic window from 590–670 nm, exhibiting exceptionally high singlet oxygen quantum yields up to 0.80. These findings illustrate that natural product PSs are highly effective agents for light-induced oxidation.

**Table 2:** Photophysical features of TCM-derived PSs.

TCM-derived PSs	Photophysical feature	
Coumarin (Zhang et al., 2024)	Absorption (λ_abs_): ~320–380 nm (peak ~350 nm) Excitation (λ_ex_): ~340–360 nm Emission (λ_em_): ~400–480 nm (blue fluorescence) Singlet oxygen quantum yield (Φ_Δ_): <0.1	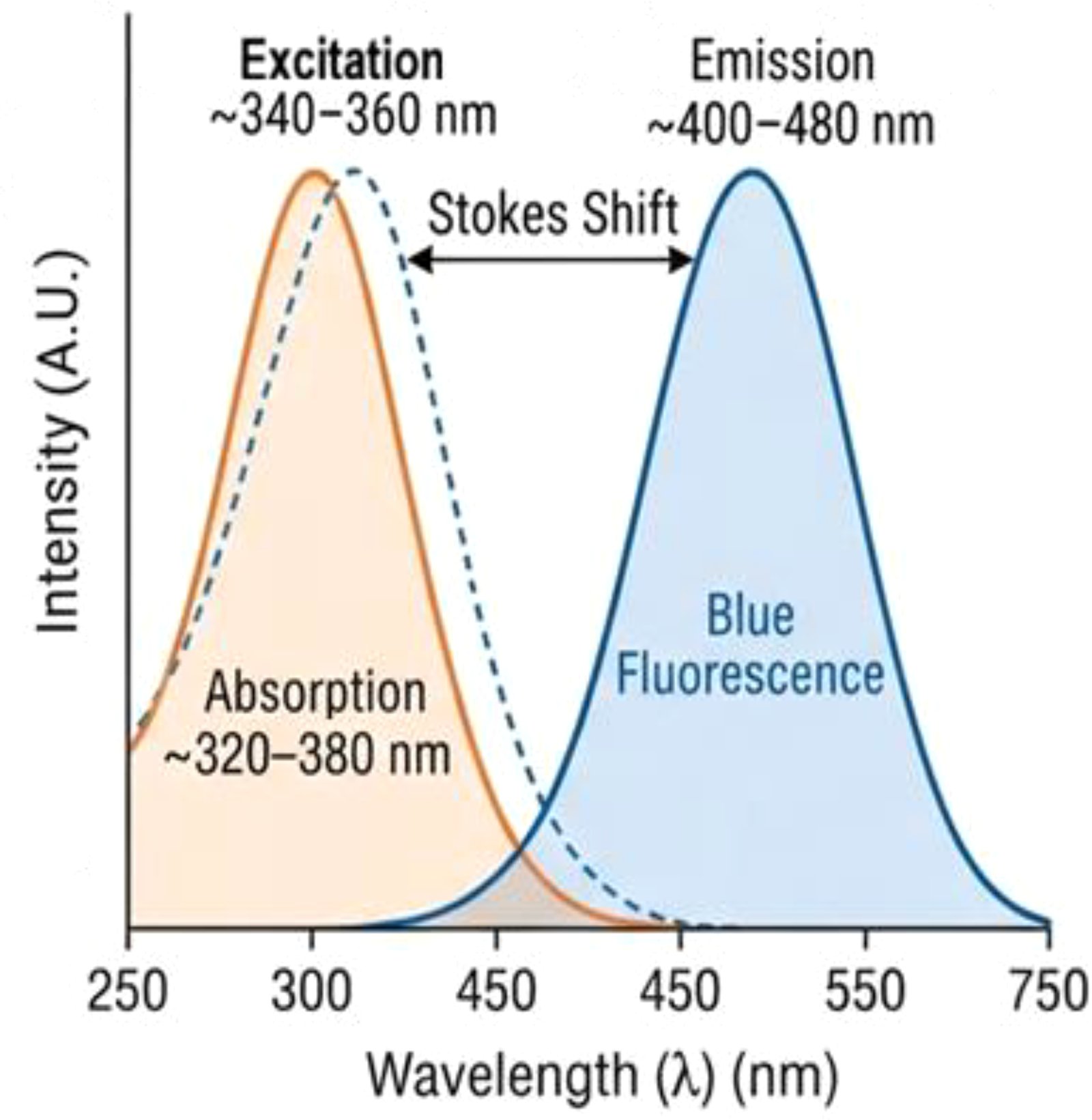
Curcumin (Gangemi et al., 2025)	Absorption (λ_abs_): ~410–430 nm Excitation (λ_ex_): ~420 nm Emission (λ_em_): ~460–550 nm Singlet oxygen quantum yield (Φ_Δ_): ~0.2–0.6	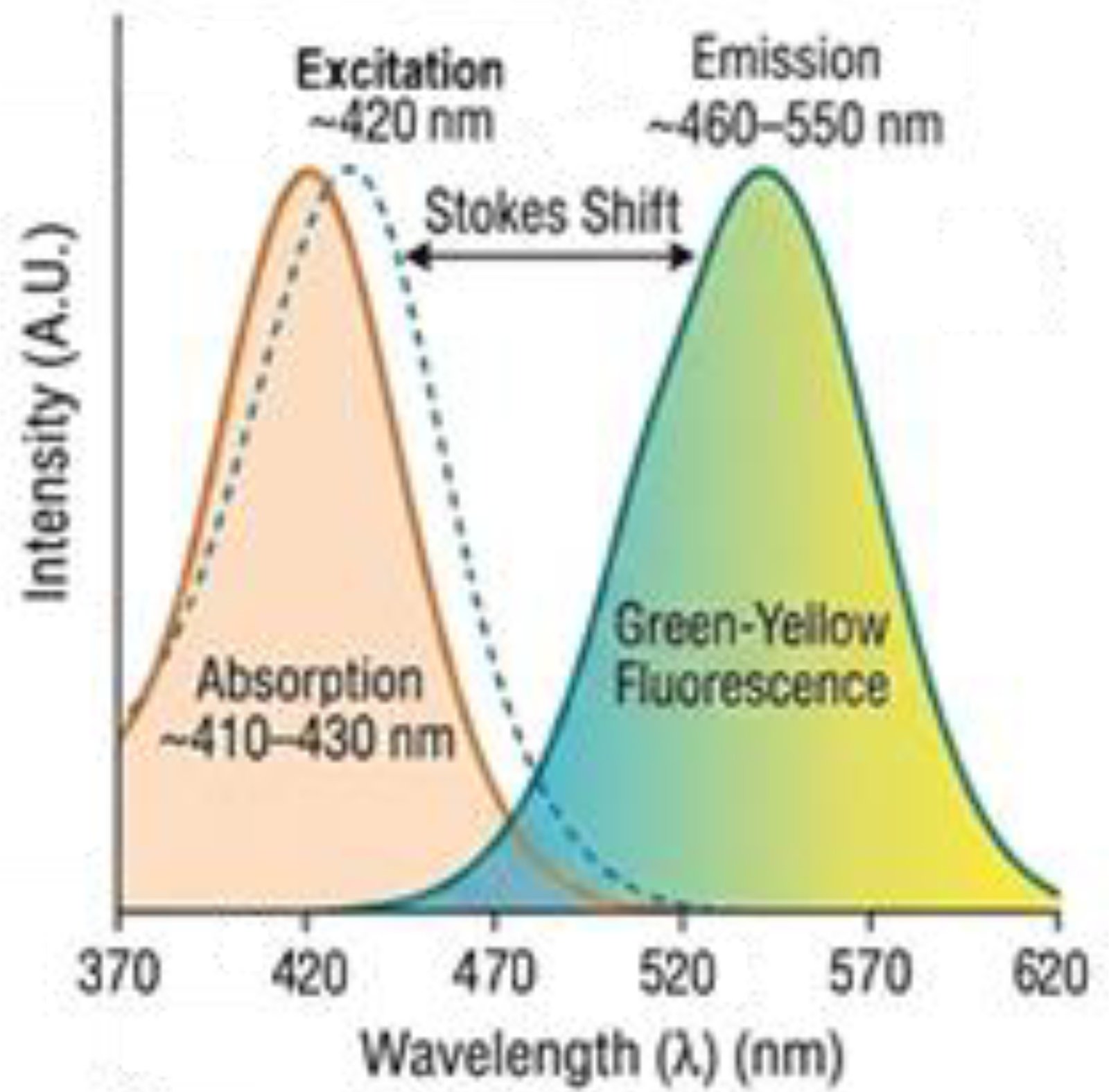
Riboflavin (Scholz et al., 2026)	Absorption (λ_abs_): 224 nm, 268 nm, 373 nm, 445 nm Excitation (λ_ex_): ~445 nm Emission (λ_em_): ~535 nm Singlet oxygen quantum yield (Φ_Δ_): ~0.54	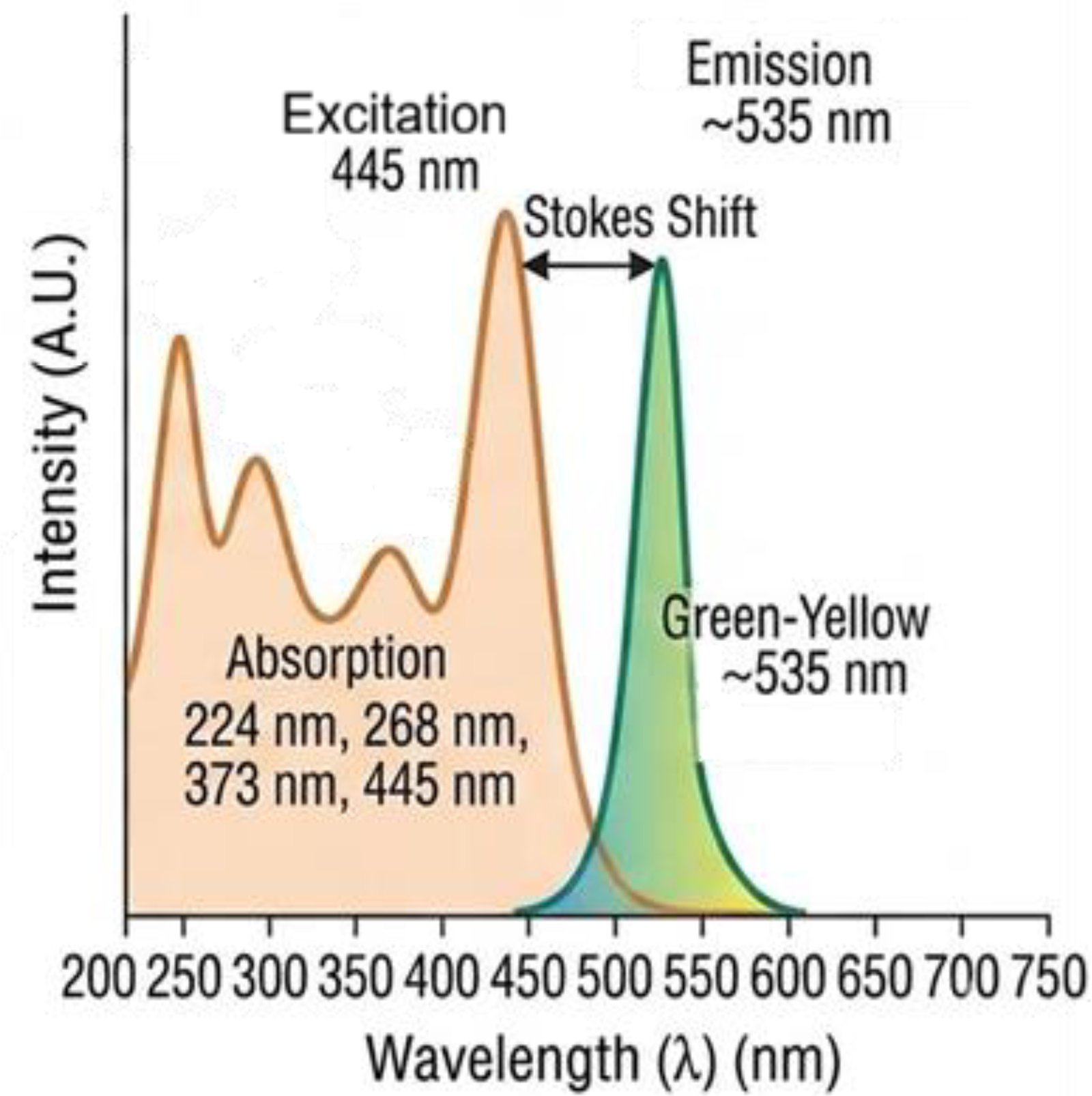
PPa (Agostinis et al., 2011)	Absorption (λ_abs_): ~413 nm (Soret band), ~667–669 nm (Q-band) Excitation (λ_ex_): ~532–670 nm Emission (λ_em_): ~675–680 nm Singlet oxygen quantum yield (Φ_Δ_): ~0.52–0.79	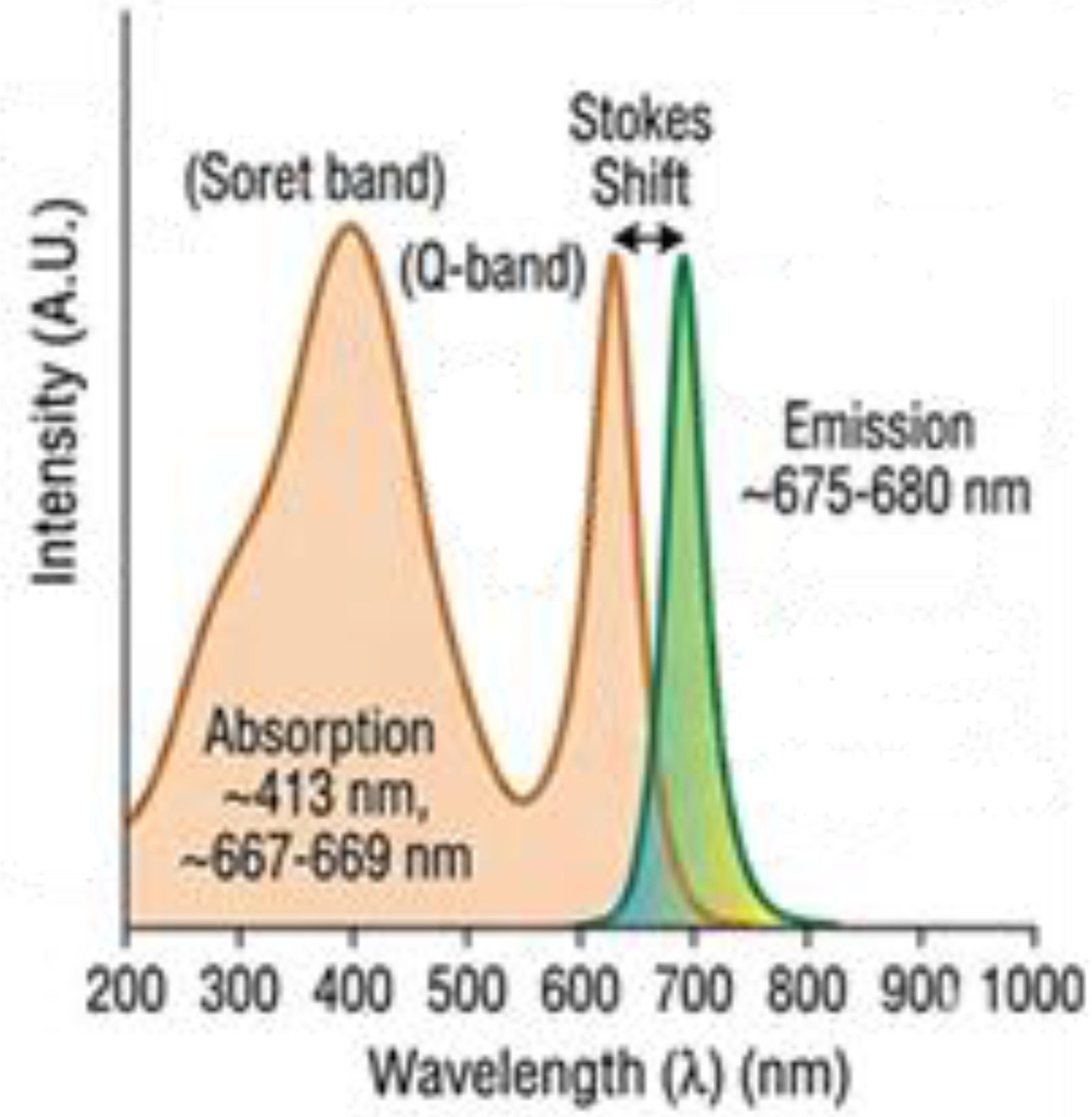
Hypericin (Lavie et al., 1995)	Absorption (λ_abs_): ~540–600 nm Excitation (λ_ex_): ~590 nm Emission (λ_em_): ~610–620 nm Singlet oxygen quantum yield (Φ_Δ_): ~0.35–0.8	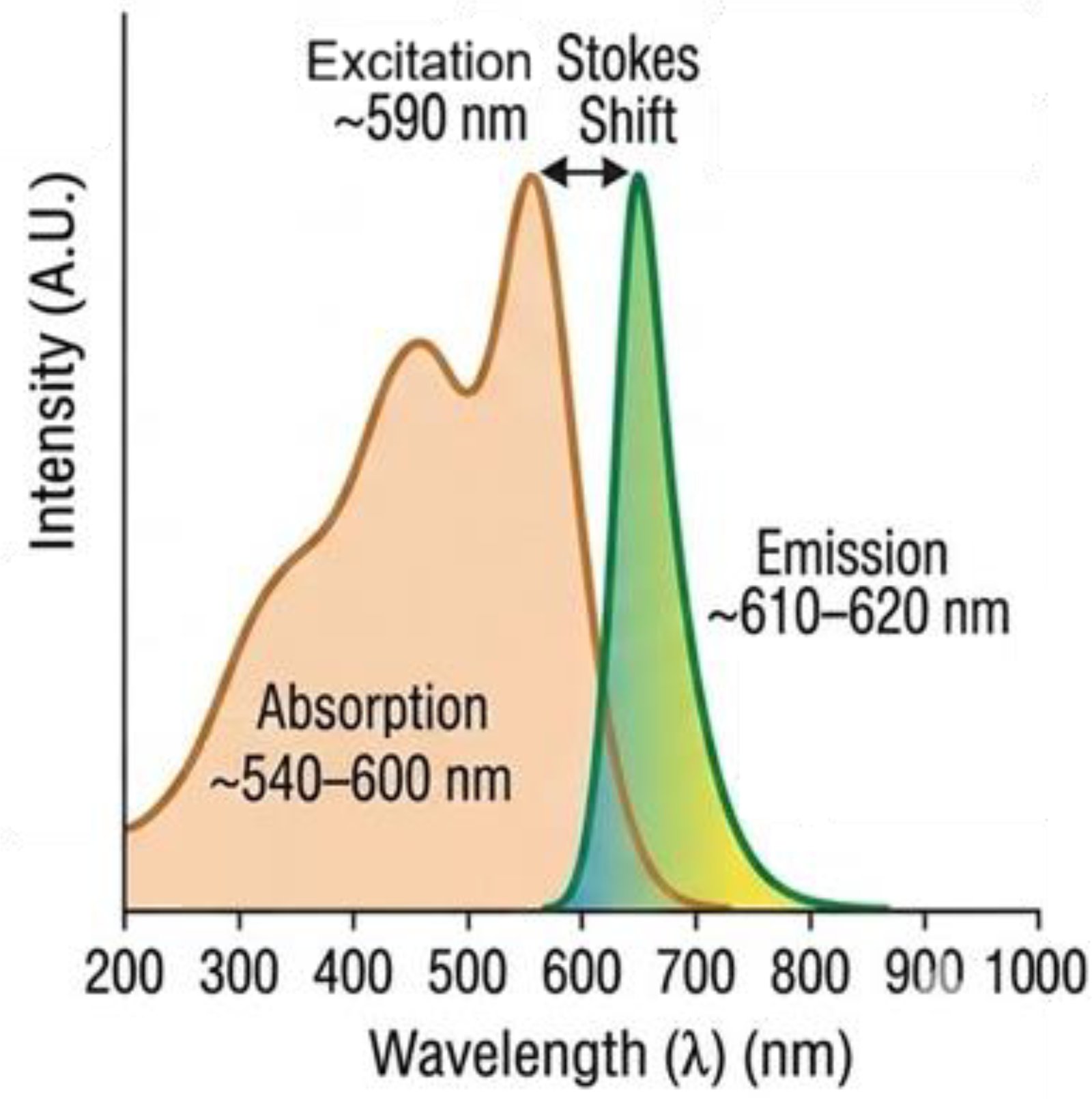

The absorption ranges span UV to deep-red for curcumin and riboflavin, which are activated by blue light, while PPa and hypericin operate in the red therapeutic window with high singlet oxygen quantum yield (Φ_Δ_). This explains TCM-derived PSs suitability for superficial compared with deep tissue infections.

## Chemical structure

The photophysical performance of TCM-derived PSs in PDT is closely linked to their chemical structure or molecular architecture. Structural frameworks such as aromatic rings, conjugated double bonds, and heteroatom substitutions dictate the electronic transitions to have the absorption (λ_abs_), excitation (λ_ex_), emission (λ_em_), singlet oxygen quantum yield (Φ_Δ_), and dual functionality. The diversity of chemical scaffolds among natural product PSs explains their distinct spectral property and behavior, which are summarized in **Table 3**.

**Table 3:** Relationship between chemical structure and photophysical performance.

TCM-derived PS	Chemical structure	Scaffold (s)
Coumarin (C_9_H_6_O_2_)	1,2-benzopyrone 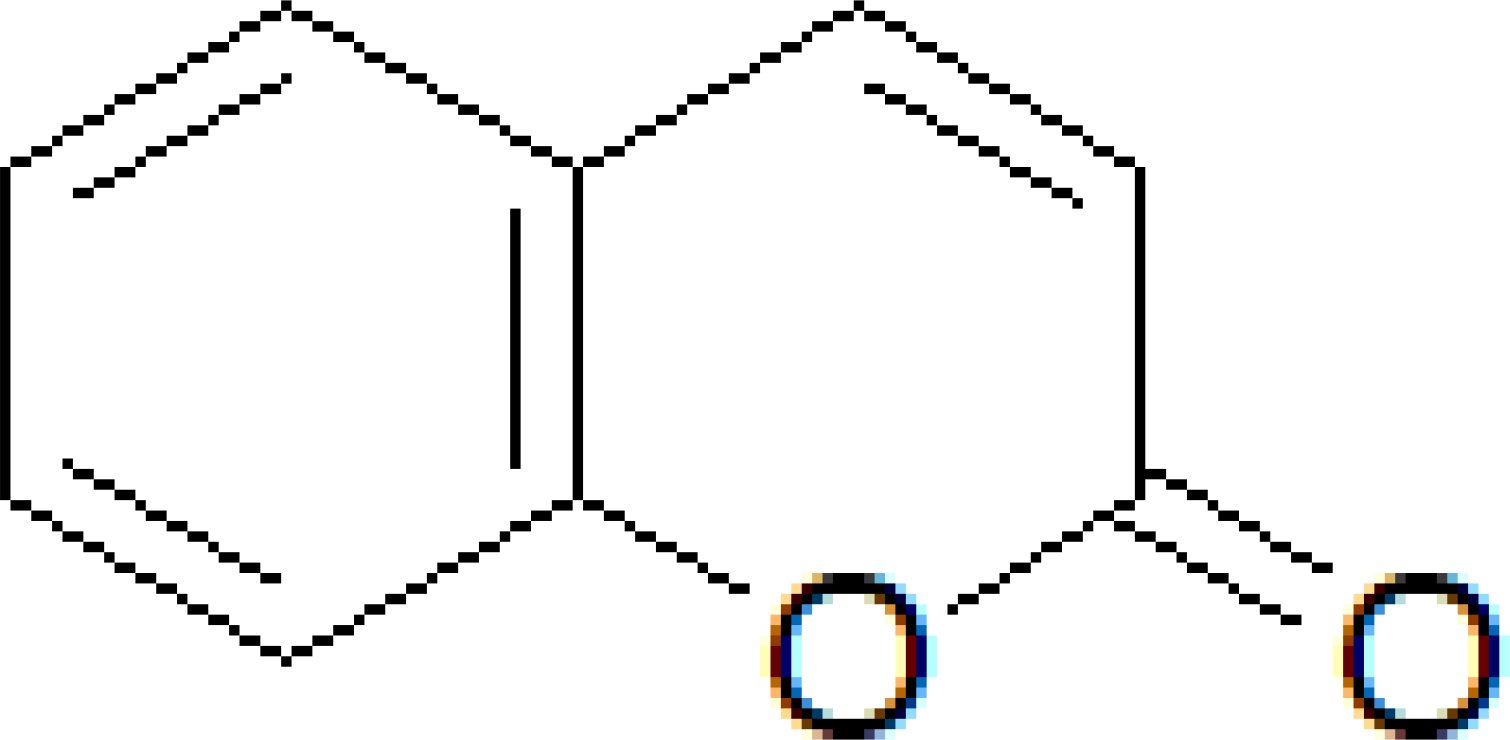	1,2-benzopyrone 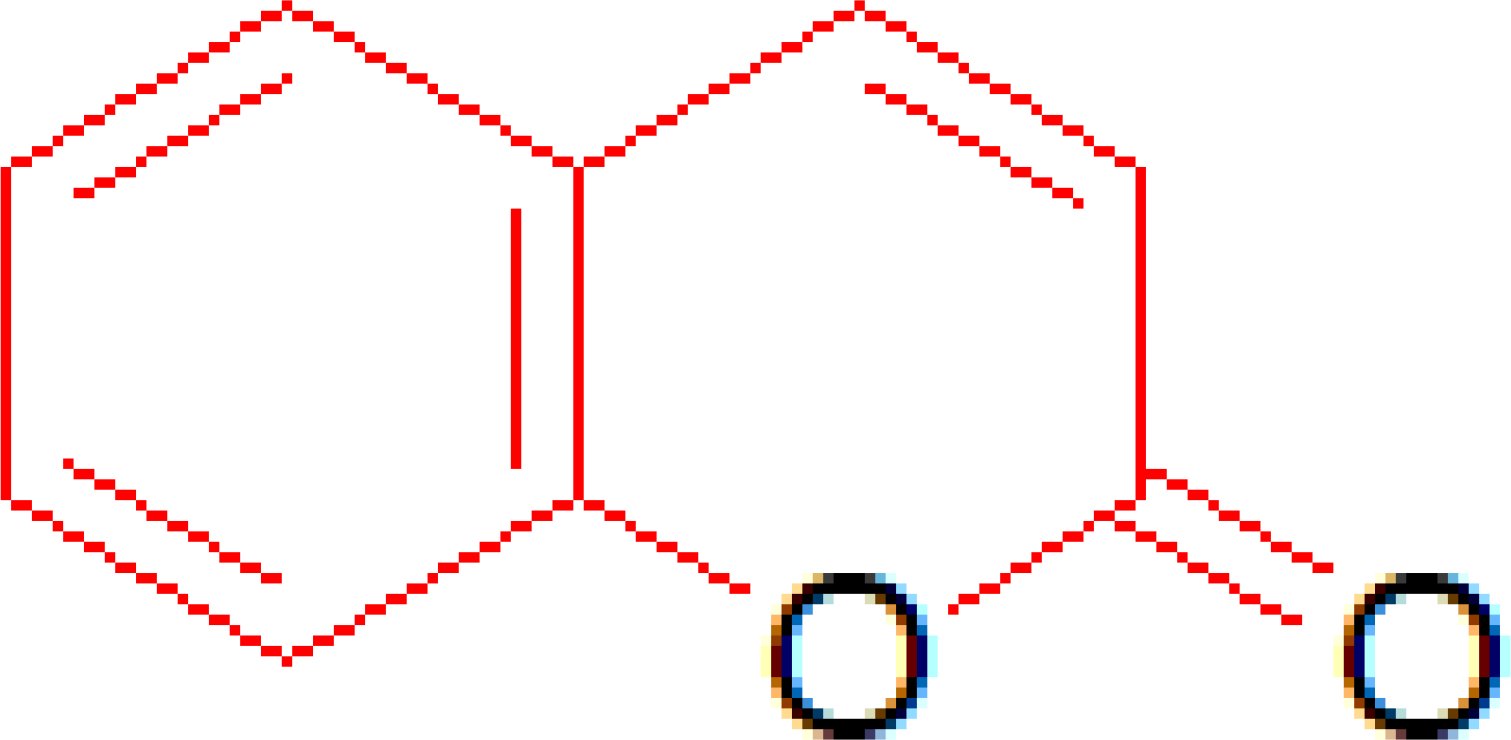
Function (s)		
1,2-benzopyrone is a backbone with a benzene ring fused to a six-membered unsaturated lactone ring (Tsivileva et al., 2022) A rigid π-conjugated framework is established to absorb UV light and emit blue fluorescence by adding electron-withdrawing groups (Jung et al., 2018)		

TCM-derived PS	Chemical structure	Scaffold (s)
Curcumin (C_21_H_20_O_6_)	(1E,6E)-1,7-bis(4-hydroxy-3-methoxyphenyl)-1,6-heptadiene-3,5-dione 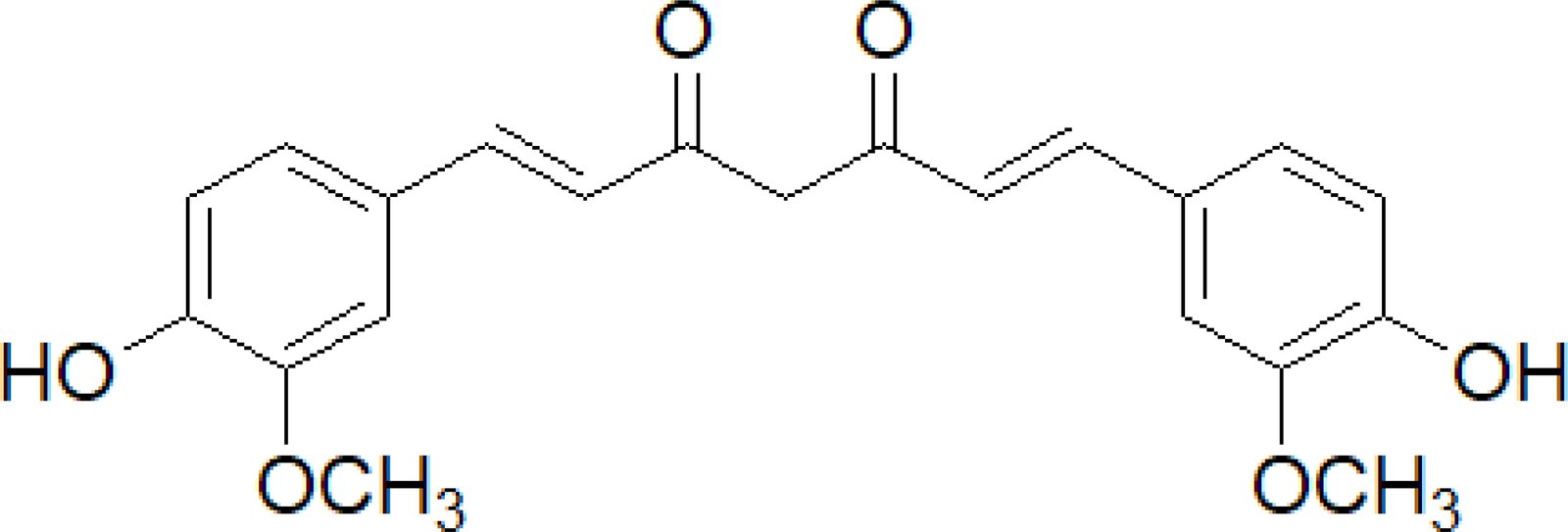	α,β-unsaturated β-diketone linker 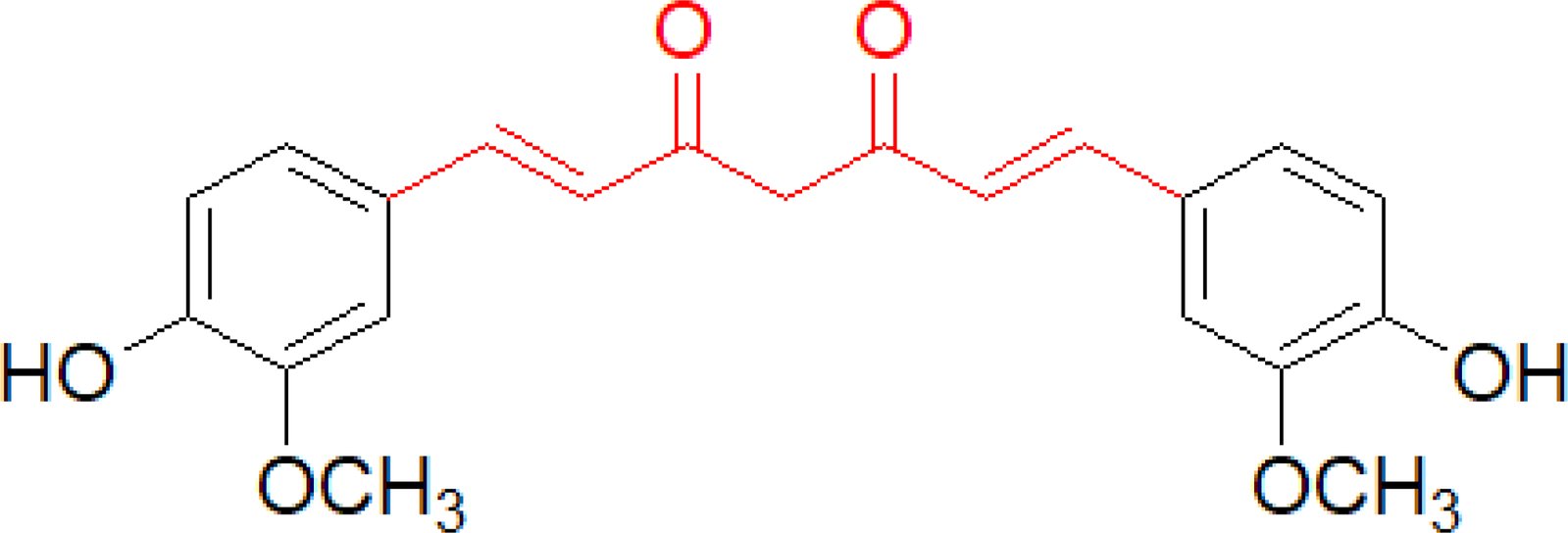 Methoxy-phenolic groups 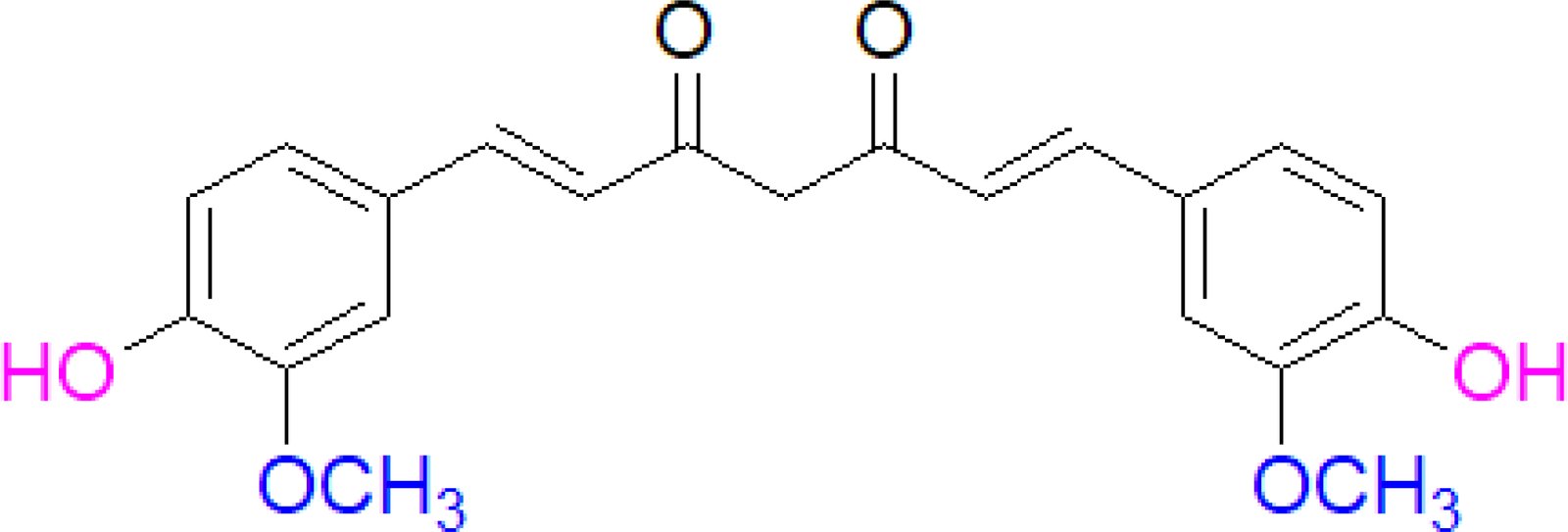 Benzene rings 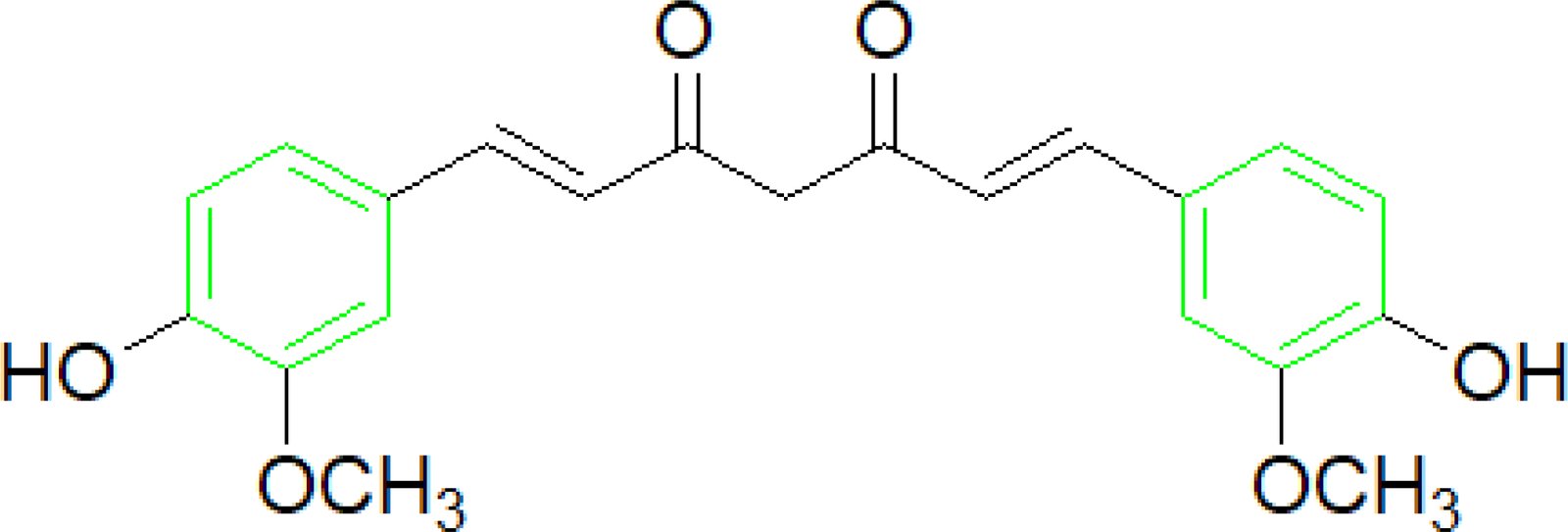
Function (s)		
The moiety of an α,β-unsaturated β-diketone linker exists in equilibrium between its diketo and keto-enol (tautomeric) forms, which modulate the photophysical properties (Nardo et al., 2009) Ortho-methoxy and phenolic hydroxyl are symmetrically placed to enhance the electron-donating capacity of the aromatic rings, significantly expanding the π-electron delocalization (Anukanon et al., 2025) An extended π-conjugation across the linker and the double bonds provides robust fluorescence and strong UV-Vis absorption in the visible range near 420 nm (López-Malo et al., 2020)		

TCM-derived PS	Chemical structure	Scaffold (s)
Riboflavin (C_17_H_10_N_4_O_6_)	7,8-dimethyl-10-(D-ribo-2,3,4,5-tetrahydroxypentyl)benzo[g]pteridine-2,4(3H,10H)-dione 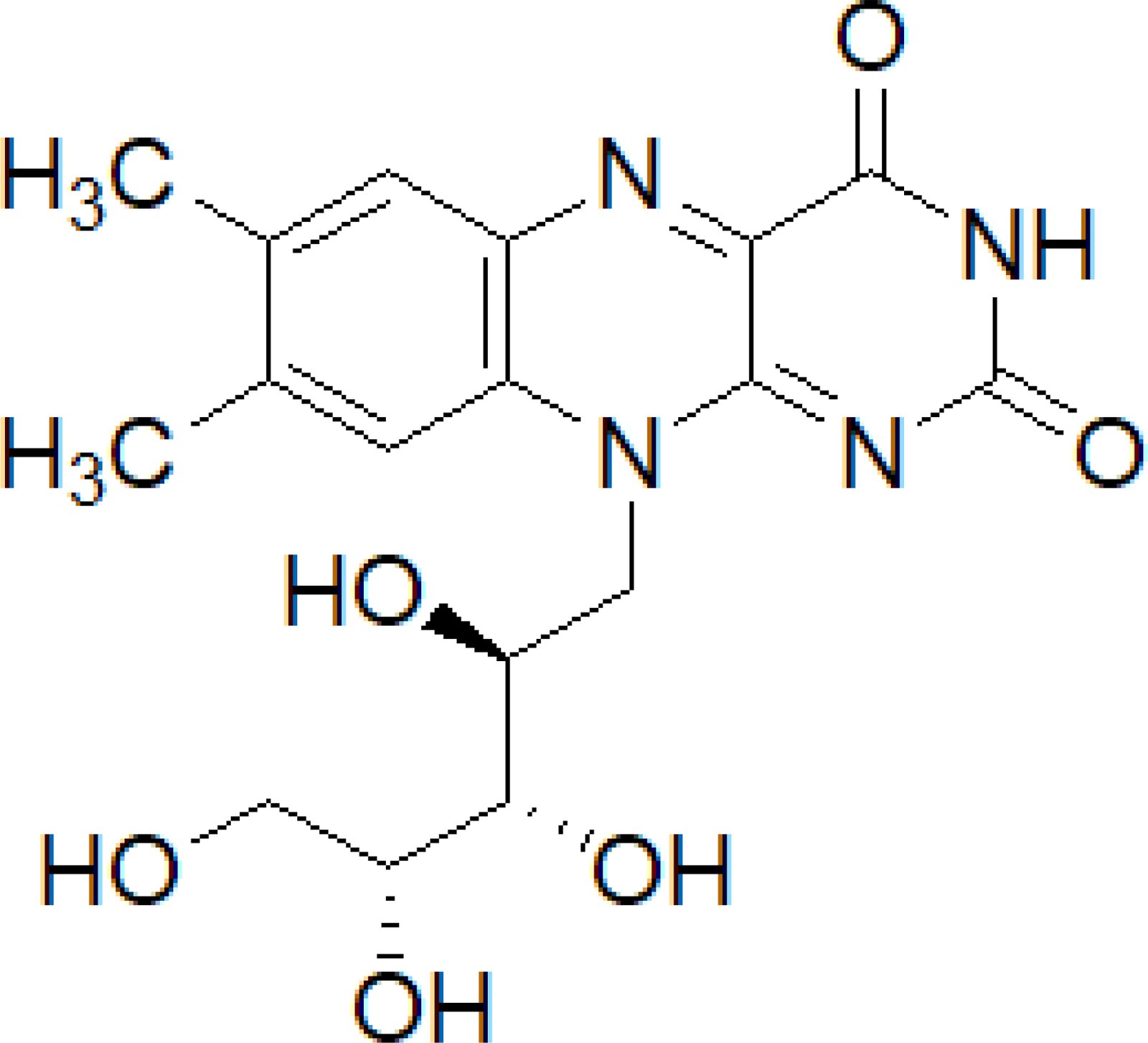	Isoalloxazine ring 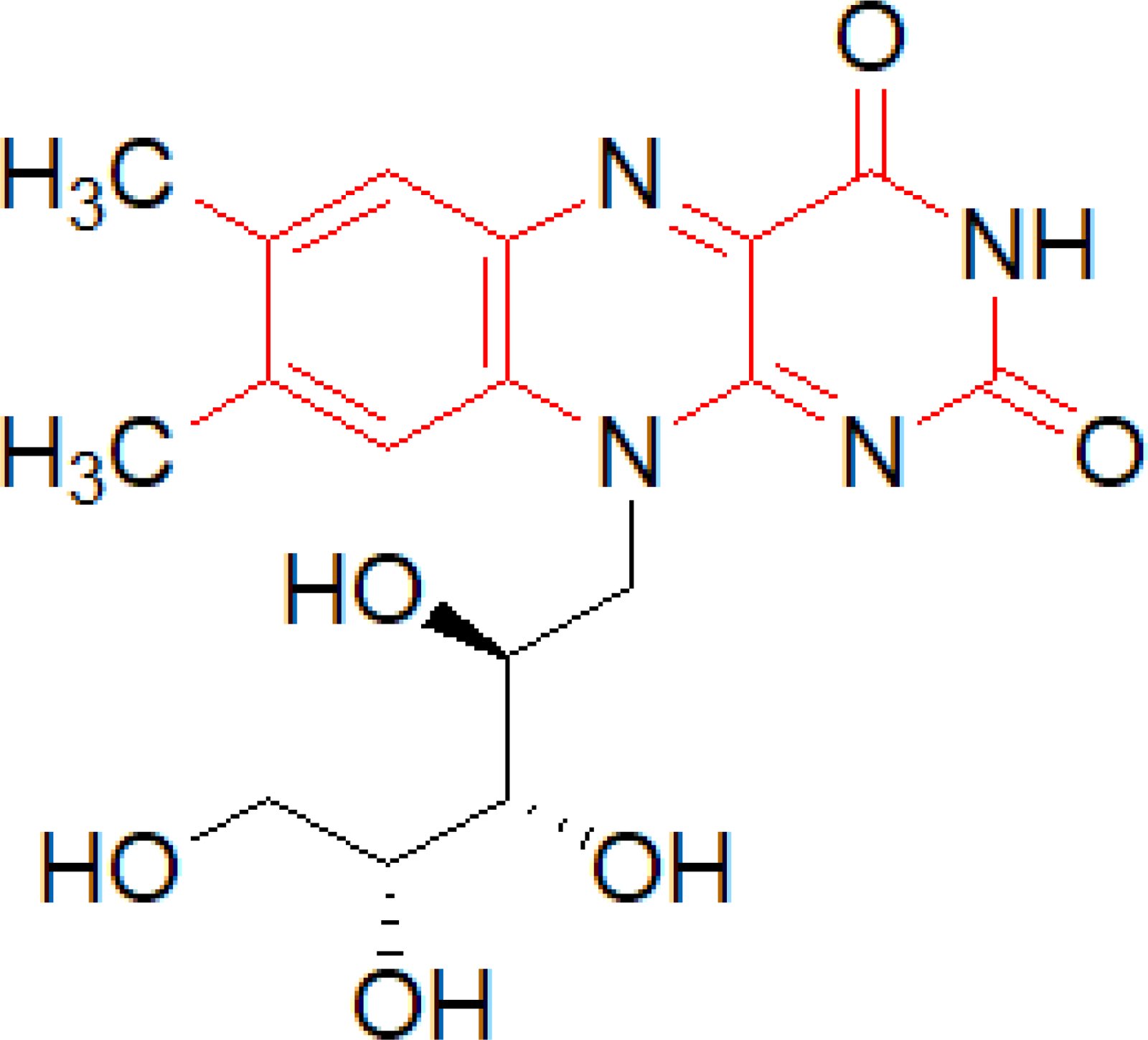 Ribityl Side Chain 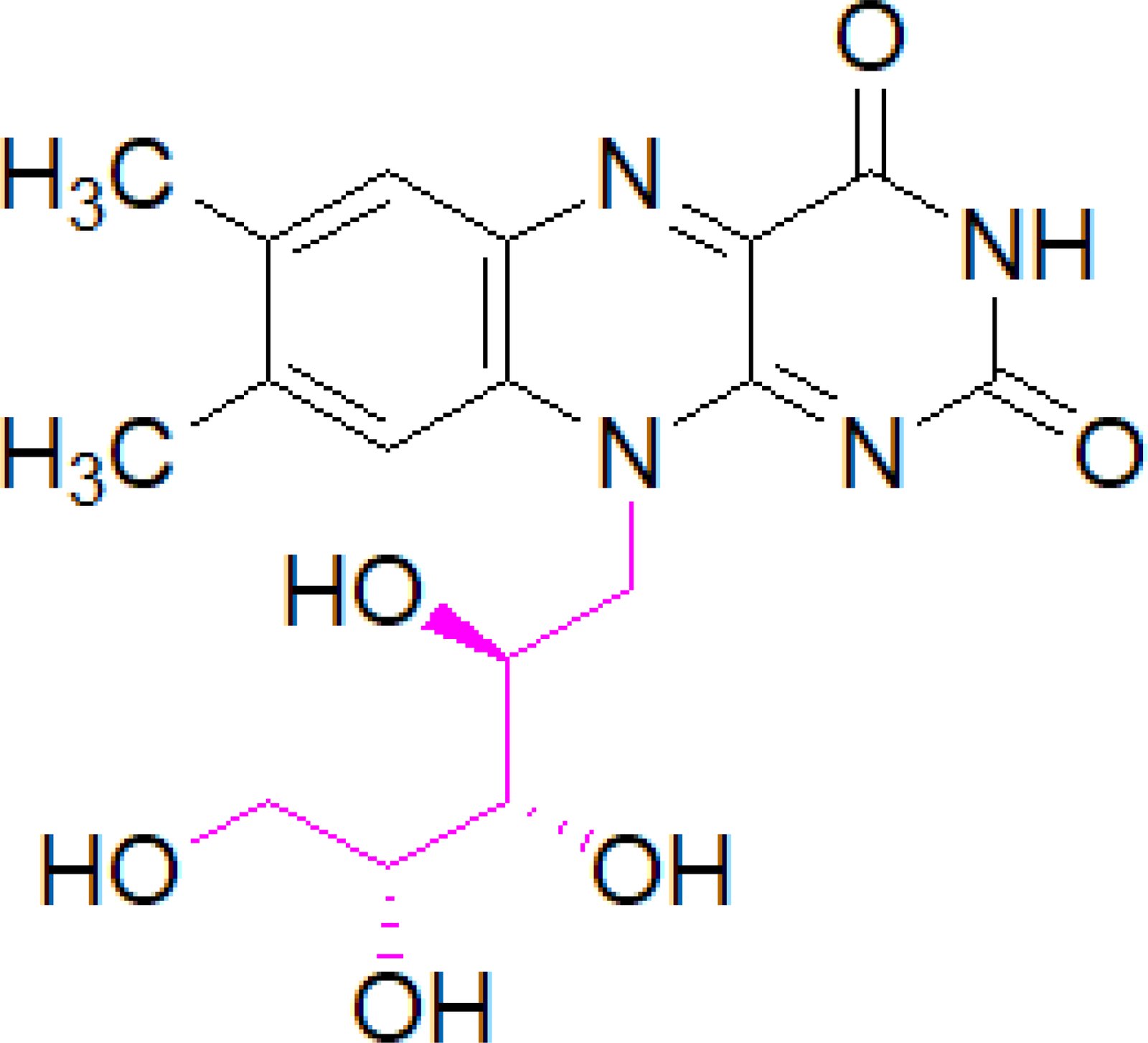
Function (s)		
The isoalloxazine ring extended π-conjugation and benzene resonance structures for its strong light absorption between 373 nm and 444 nm with characteristic intense yellow-green fluorescence under UV light (Sheraz et al., 2014) The hydrophilic polyhydroxy group of a ribityl side chain increases the water solubility and dictates the interaction with the microenvironment, leading to better absorption (Rich et al., 2014)		

TCM-derived PS	Chemical structure	Scaffold (s)
PPa (C_33_H_34_N_4_O_3_)	9-ethenyl-14-ethyl-4S,8,13,18-tetramethyl-20-oxo-3S-phorbinepropanoic acid 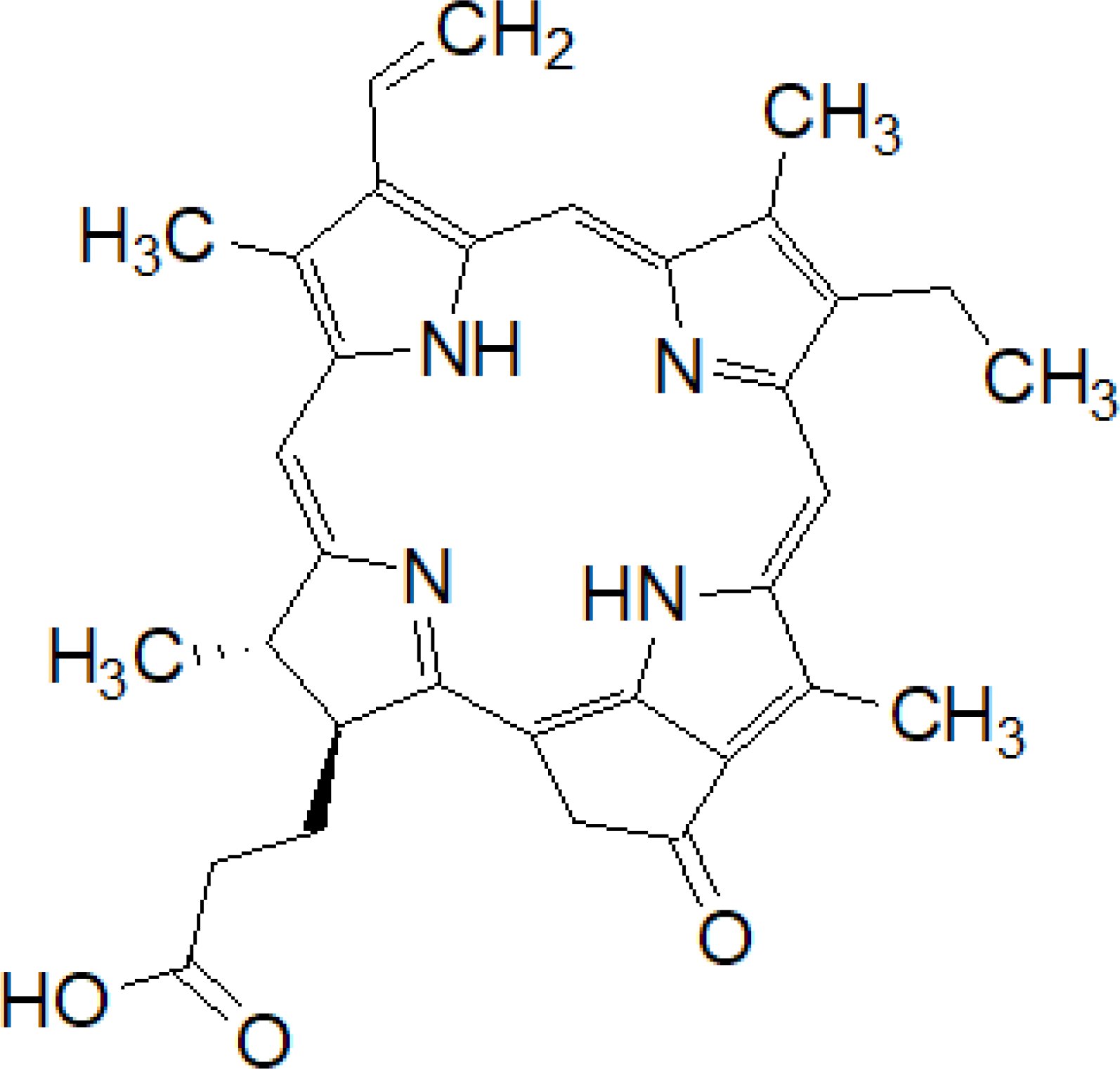	Porphyrin core 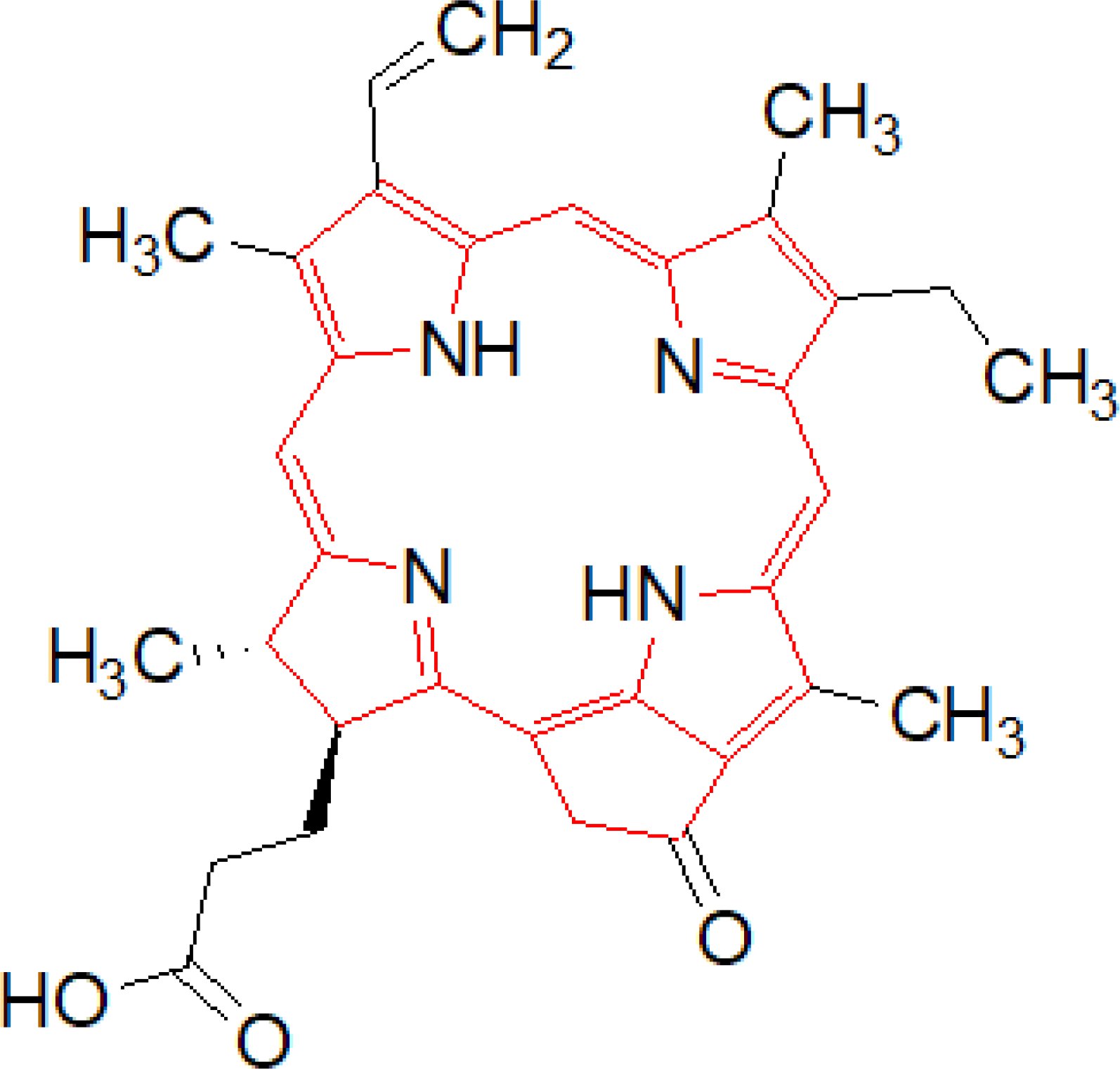 Functional acid and vinyl groups 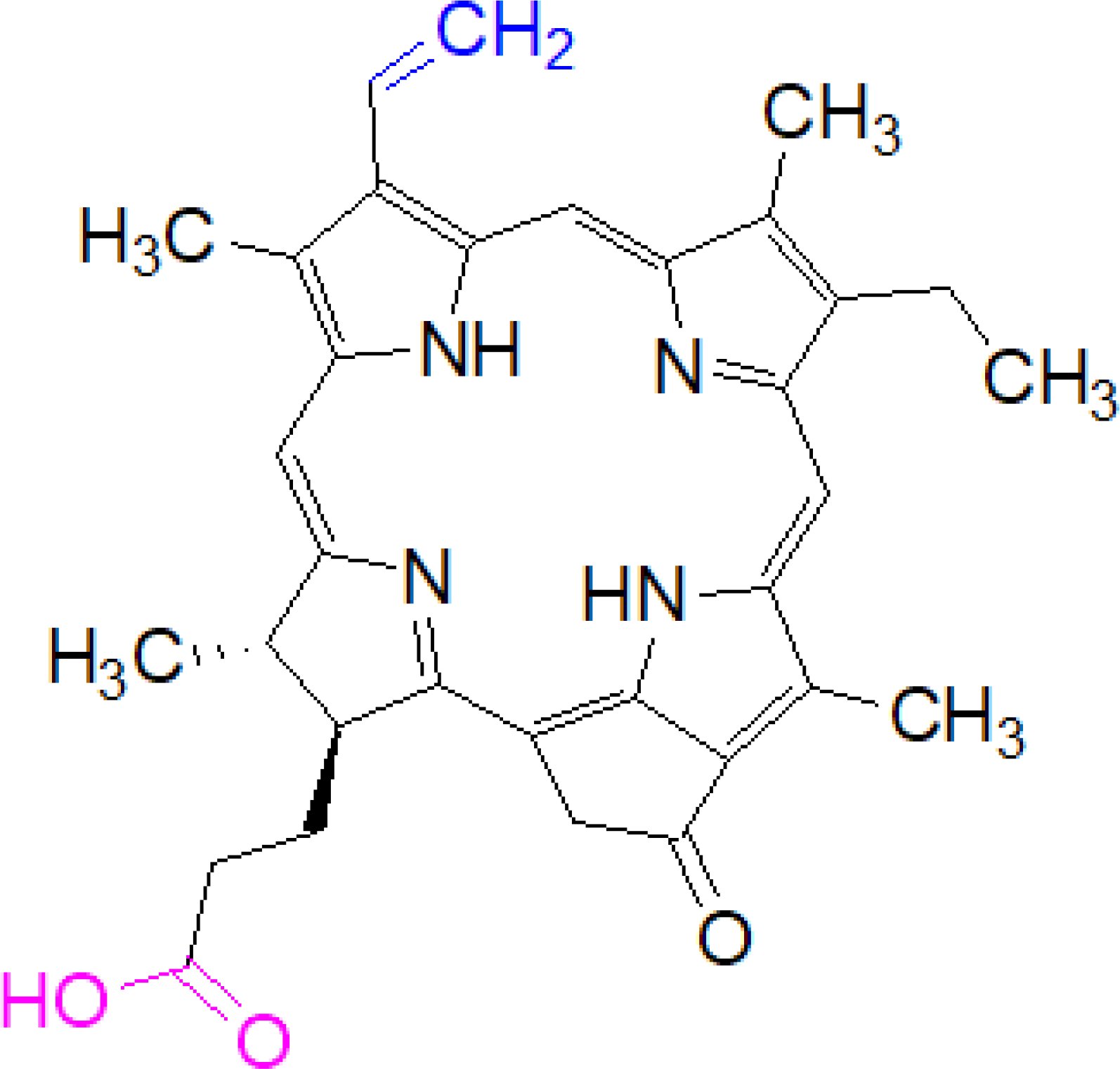
Function (s)		
The conjugate π-electron network enhances its photophysical behavior, allowing for deep-red light absorption (Myrzakhmetov et al., 2021) A propanoic acid group defines its amphiphilic nature, and the vinyl group fused cyclopentanone ring allows for targeted chemical modifications to alter pharmacokinetics and the photophysical properties (Pandey et al., 2009; Marko et al., 2019)		

TCM-derived PS	Chemical structure	Scaffold (s)
Hypericin (C_30_H_16_O_8_)	1,3,4,6,8,13-Hexahydroxy-10,11-dimethylphenanthro[1,10,9,8-opqra]perylene-7,14-dione 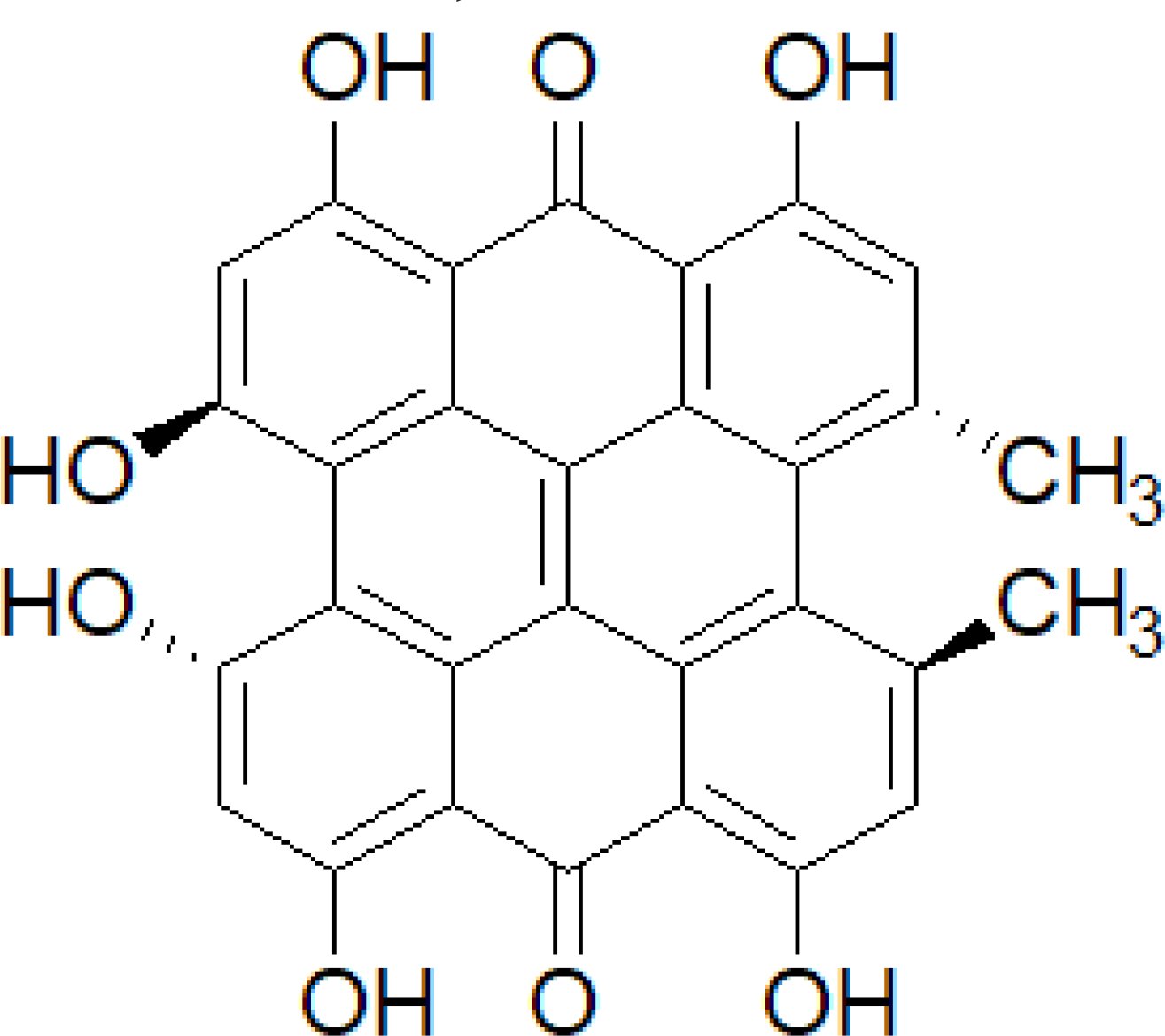	Extended π-conjugation 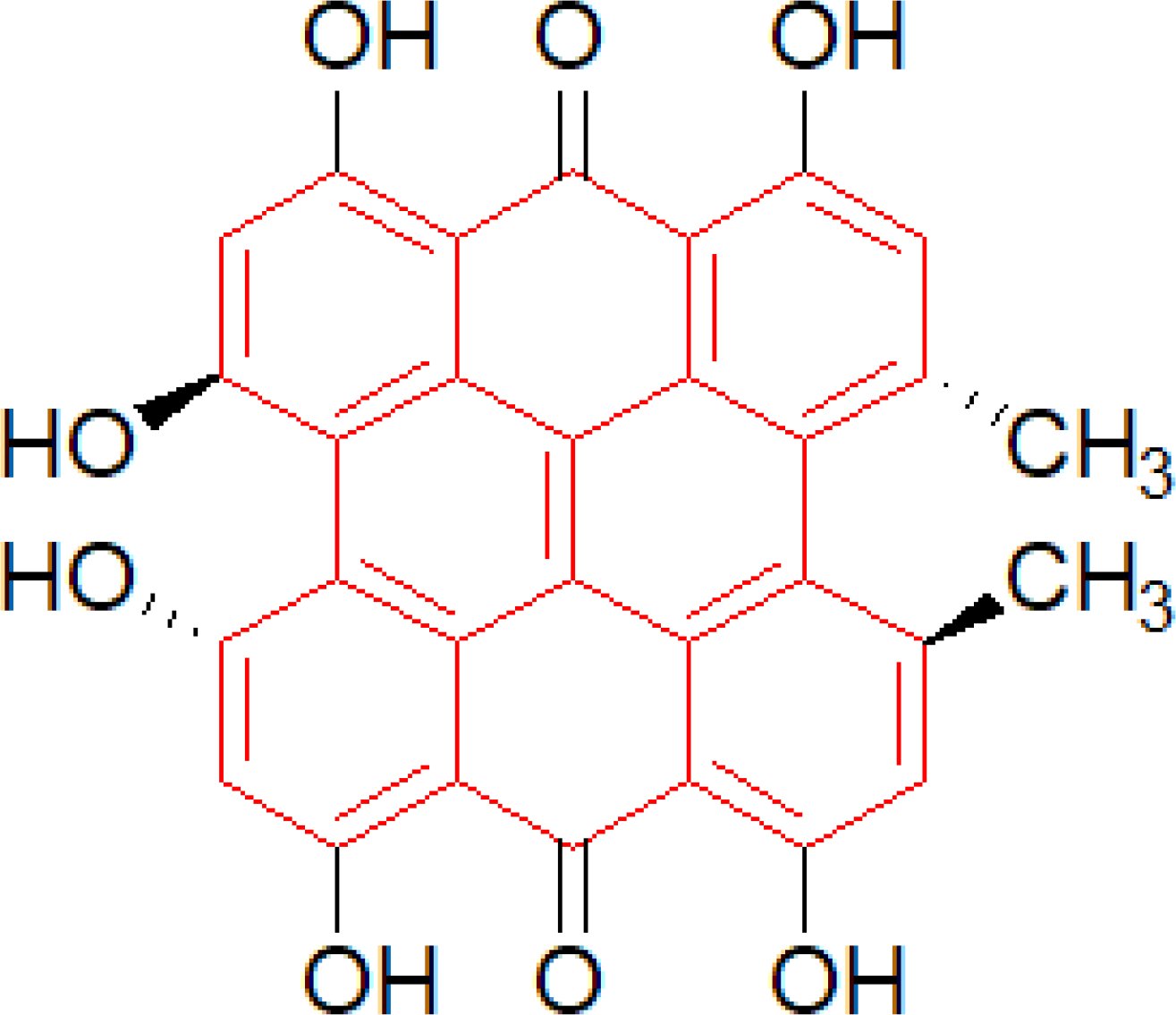 Perihydroxyl groups 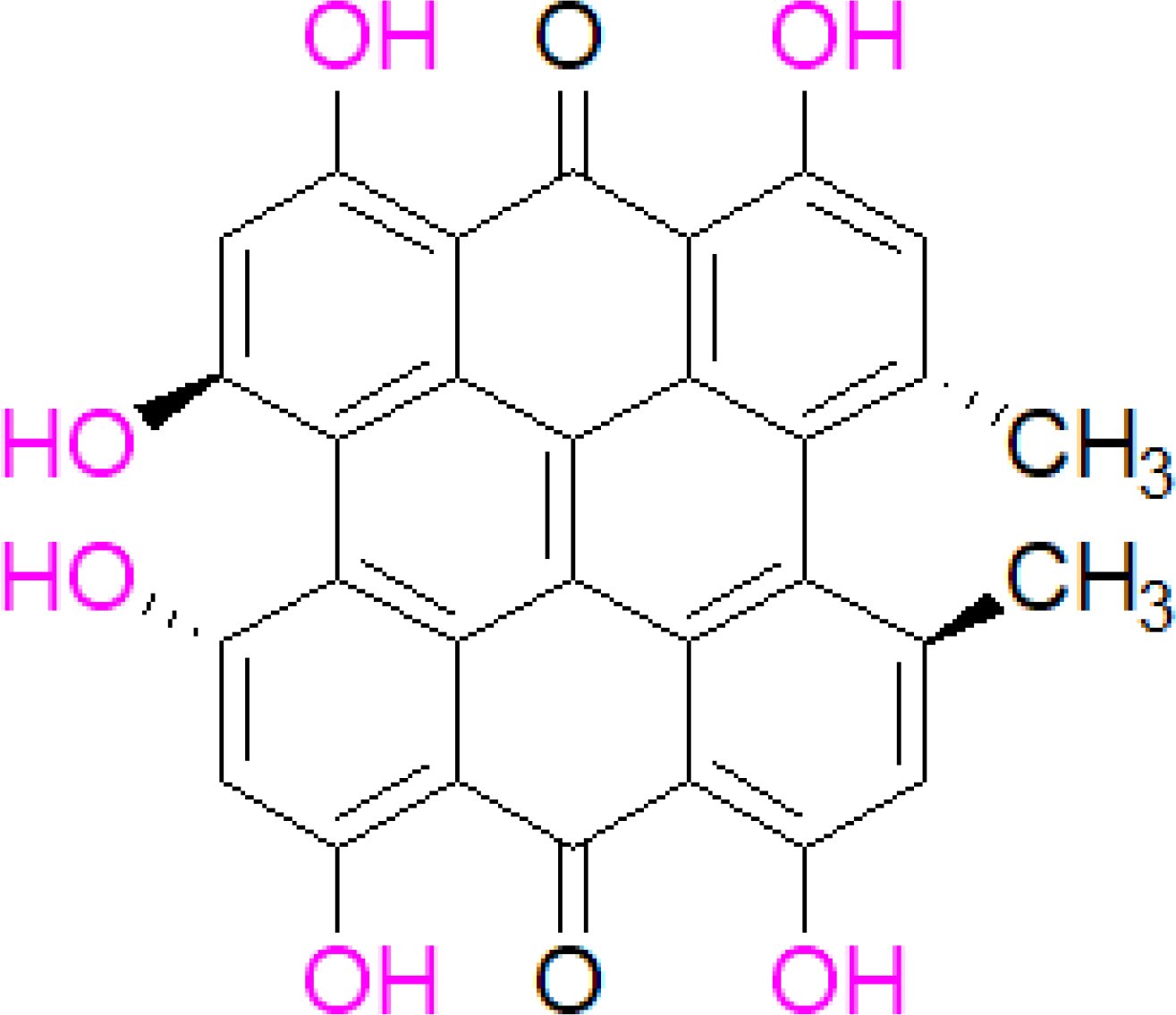
Function (s)		
The core consists of a symmetrical, heavily conjugated, fused polycyclic aromatic system to provide extra stability and determine for its visible-light absorption (Gattuso et al., 2017) This contains highly reactive phenolic hydroxyl groups, which are prone to forming strong intramolecular hydrogen bonds, which stabilize the molecule and influence its acid-base equilibrium for the better generation of ROS (Uličný and Laaksonen, 2020)		

Explain the structural scaffolds, such as benzopyrone, diketone linker, isoalloxazine ring, porphyrin core, and polycyclic aromatic system. These dictate absorption spectra, ROS yield, solubility, and emphasize that the porphyrin-based PPa and hypericin provide superior photophysics but require delivery optimization.

In summary, the chemical scaffolds of TCM-derived PSs critically determine their photophysical behavior, ROS generation, and therapeutic applicability. Coumarin’s rigid benzopyrone ring absorbs UV light, but yields limited singlet oxygen, restricting PDT potency compared with porphyrin-based agents. Curcumin’s extended π-conjugation and keto-enol tautomerism enhance visible-light absorption and ROS generation, but its instability and poor solubility hinder systemic use. Riboflavin’s isoalloxazine ring provides strong absorption in the blue region and efficient singlet oxygen yield, and its dependence on oxygen availability reduces efficacy in hypoxic infections. In contrast, PPa possesses a porphyrin core with deep-red absorption and high quantum yield, enabling superior tissue penetration. Hypericin’s polycyclic aromatic system and reactive hydroxyl groups confer high ROS production but raise concerns of phototoxicity. These structural distinctions explain the variability in antimicrobial spectrum and translational potential, highlighting the need for chemical modifications and nanocarrier formulations to optimize clinical performance.

## PDT mechanism

Generally, PDT requires three major components including PS, light with a specific wavelength, and molecular oxygen. PS absorbs light energy, transits to an excited state, and transfers energy to surrounding oxygen molecules, producing ROS, such as singlet oxygen (^1^O_2_), superoxide (O_2_^•−^), and hydroxyl radicals (•OH). These ROS are highly cytotoxic and initiate oxidative stress within microbial cells, and the damage occurs at multiple levels, such as lipid peroxidation disrupting cell membranes, protein oxidation inactivating enzymes, and DNA fragmentation impairing replication and repair. This multi-target oxidative assault bypasses conventional resistance mechanisms, leading to the PDT especially valuable against multidrug-resistant pathogens (Demidova and Hamblin, 2004; Kadbhane et al., 2025).

PDT eliminates pathogens by having two complementary photochemical pathways known as Type I and Type II mechanisms. Type I pathway involves the excited triplet state of the PS undergoing electron or hydrogen transfer reactions with nearby biomolecules, producing free radicals such as O_2_^•−^, •OH, and H_2_O_2_. These reactive intermediates initiate chain reactions to damage lipids, proteins, and nucleic acids, amplifying oxidative stress within microbial cells. In contrast, the Type II pathway involves direct energy transfer from the photosensitizer’s triplet state to molecular oxygen, yielding ^1^O_2_, which is a highly reactive species that oxidizes unsaturated lipids and amino acid side chains, causing irreversible structural modifications. The balance between these two routes depends on oxygen availability and the biochemical microenvironment. Type II singlet oxygen dominates under normoxic conditions, while hypoxic settings favor Type I radical processes to ensure PDT maintains antimicrobial efficacy across diverse infection sites (Ding et al., 2011; Huang et al., 2012) (**Figure 2**).

**Figure 2:**
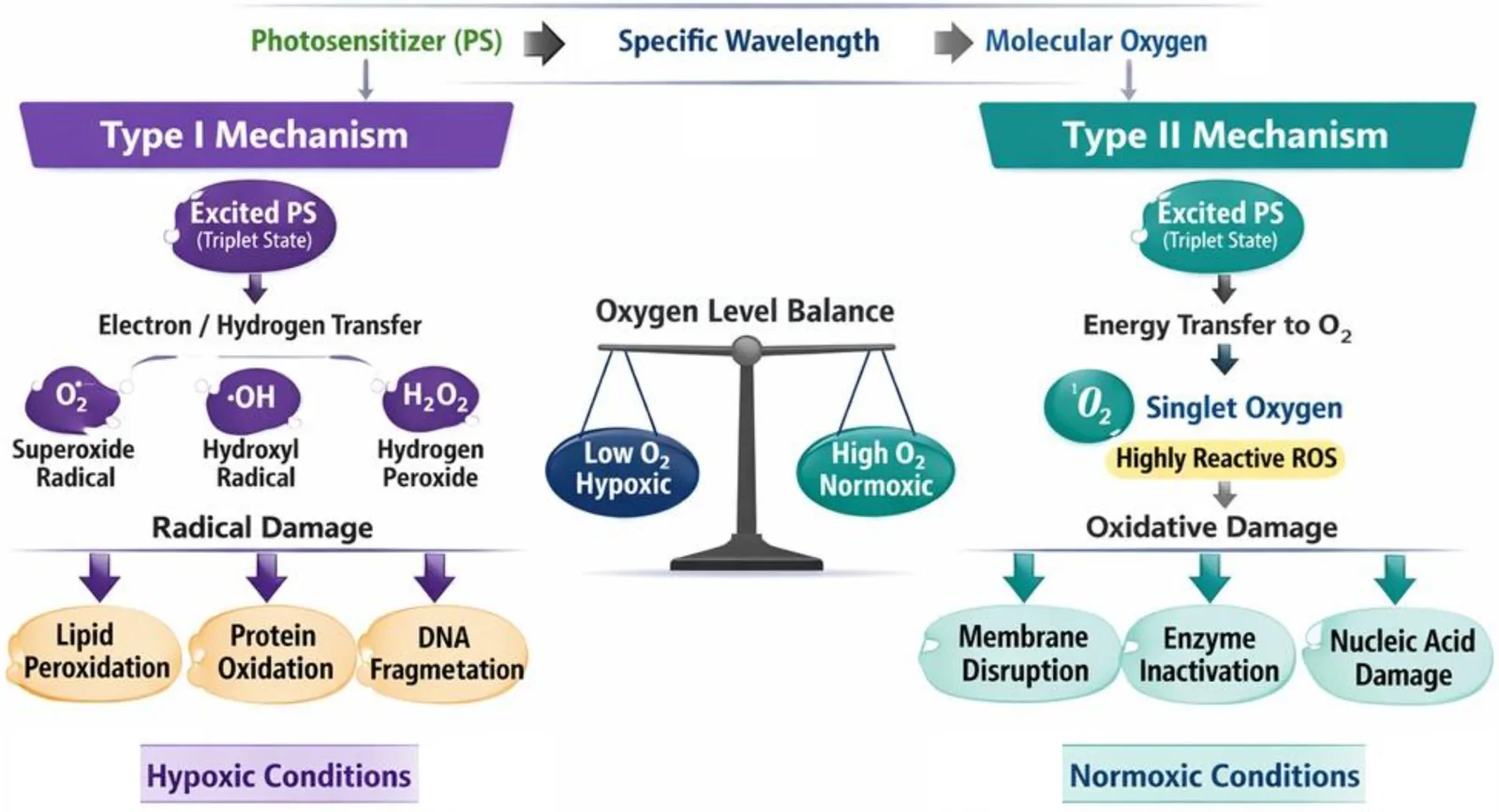
PDT mechanism for type I and type II pathways.

The more detailed categorization of ROS clarifies their distinct roles in antimicrobial activity. ^1^O_2_ is the hallmark cytotoxic species in Type II PDT, which reacts rapidly with unsaturated lipids and amino acid side chains, destabilizing microbial membranes and inducing irreversible protein oxidation (Demidova and Hamblin, 2004). O_2_^•−^ is generated through Type I electron transfer that initiates secondary radical cascades and undergoes dismutation to form H_2_O_2_, which diffuses across membranes and amplifies oxidative stress (Huang et al., 2012). •OH derived from fenton-type reactions of H_2_O_2_ are highly reactive that cause direct DNA strand breaks, base modifications, and cross-linking, thereby impairing replication and repair (Ding et al., 2011). These ROS act at multiple levels, including lipid peroxidation to compromise membrane integrity, protein oxidation inactivates enzymes and structural proteins, and nucleic acid damage disrupts genetic stability. The multi-target oxidative assault bypasses conventional resistance mechanisms, explaining PDT’s efficacy against multidrug-resistant pathogens.

TCM-derived advanced PSs in PDT provide an innovative antimicrobial strategy by harnessing light-activated oxidative stress. The natural compound contributes unique photophysical properties to enhance pathogen eradication and align with ethnopharmacological traditions.

Coumarin is a versatile PS in PDT by engaging both Type I and Type II mechanisms, which enter an excited triplet state upon light activation. Type I pathway undergoes electron or hydrogen transfer reactions, producing free radicals such as O_2_^•−^ and •OH to damage microbial membranes and proteins. Type II pathway generates ^1^O_2_, which oxidizes lipids, proteins, and nucleic acids, leading to irreversible cellular damage. This dual mechanism ensures broad oxidative stress, simultaneously disrupting cell walls and genetic material, thereby enhancing antibacterial efficacy even against resistant strains. Meanwhile, the structural adaptability allows fine-tuning of its absorption wavelength, improving compatibility with clinical light sources and expanding therapeutic applications (Ma et al., 2024).

Curcumin’s antibacterial activity against *S. aureus* in PDT is mainly driven by the Type I mechanism. This reaches an excited triplet state when exposed to blue light and undergoes electron transfer reactions, producing ROS, which attack bacterial cell walls through lipid peroxidation, impair metabolic enzymes by oxidizing thiol groups, and destabilize biofilms by degrading extracellular polymeric substances. More importantly, it remains effective under hypoxic conditions, as Type I reactions do not rely solely on oxygen availability. This dual role as a natural antioxidant and PS enhances therapeutic selectivity, allowing potent pathogen destruction while protecting host tissues from excessive oxidative stress (Yuan et al., 2022).

Riboflavin PDT primarily operates through the Type II mechanism, although the Type I pathways may occur depending on the environment. When riboflavin absorbs light, it transitions to an excited triplet state and efficiently transfers energy to molecular oxygen, producing ^1^O_2_. This highly reactive species oxidizes lipids, proteins, and nucleic acids, leading to widespread microbial inactivation. It has a relatively high Φ_Δ_, its antimicrobial efficacy is strongly linked to the Type II mechanism. However, under conditions of reduced oxygen availability, it can engage in Type I reactions, generating free radicals through electron transfer. This dual capacity enhances its versatility, but the dominant pathway in normoxic environments is Type II singlet oxygen production, which explains its effectiveness against bacteria and fungi while maintaining biocompatibility and nutritional relevance (Crocker et al., 2022; Huang et al., 2012).

Hypericin and PPa are natural pigments that act as PSs in PDT against methicillin-resistant *S. aureus* (MRSA). These compounds enter an excited triplet state upon light activation and efficiently transfer energy to molecular oxygen, producing ^1^O_2_ with a high quantum yield. This Type II mechanism is the dominant pathway, as singlet oxygen rapidly oxidizes lipids, proteins, and nucleic acids, leading to irreversible bacterial damage and cell death. Translational studies have confirmed their efficacy, showing consistent results from *in vitro* assays to *in vivo* wound infection models. PPa’s structure enhances tissue penetration, broadening PDT’s therapeutic scope, while hypericin’s strong ROS generation ensures rapid microbial killing, underscoring their clinical relevance (Chan et al., 2021).

Beyond the Type I and Type II mechanisms, photothermal effects should also be considered during the PDT process, particularly when natural product PSs are employed. Curcumin and hypericin exhibit strong absorption in the visible to near-infrared region, which can generate localized heat under continuous irradiation, thereby enhancing microbial eradication by disrupting cell membranes, denaturing proteins, and weakening biofilm matrices, thereby complementing ROS-mediated cytotoxicity (Agostinis et al., 2011; Gattuso et al., 2017). The synergistic interplay between photodynamic and photothermal mechanisms is especially relevant in hypoxic infection sites, where oxygen limitation reduces ^1^O_2_ and radical pathways dominate. However, excessive photothermal heating may increase the risk of collateral tissue damage and phototoxicity, underscoring the importance of optimizing irradiation parameters, PS concentration, and nanocarrier formulations (Marko et al., 2019; Uličný and Laaksonen, 2020). Integrating photothermal considerations into PDT design provides a more nuanced understanding of natural product-based therapies and supports their translational potential in the measurement of infection control for pathogens.

## Research process

The photophysical properties and mechanisms of TCM-derived PSs in PDT have been established by evaluating the therapeutic potentials against pathogenic infections, including antimicrobial, antiviral, antifungal, and antiparasitic effects. For instance, coumarin-based probes demonstrated strong antimicrobial efficacy under 660 nm near-infrared irradiation for the generation of ROS that eradicated *S. aureus* while enabling deep-tissue imaging (Li et al., 2022). Modified coumarin derivatives further enhanced the dual Type I/II ROS generation to improve antibacterial outcomes. Curcumin indicated potent activity against *S. aureus* biofilms through blue-light activation, while riboflavin exhibited broad-spectrum antimicrobial effects linked to ^1^O_2_ production (Crocker et al., 2022). PPa and hypericin operated in the red-light therapeutic window, achieved high quantum yields, and effectively targeted methicillin-resistant *S. aureus* and fungal pathogens (Chan et al., 2021). These examples highlight the translational potential of TCM-derived PSs, combining intrinsic pharmacological activity with photodynamic effects to overcome resistance and broaden therapeutic applications (**Table 4**).

**Table 4:** Current representative examples of TCM-derived PSs in PDT.

Topic	TCM-derived PS	PDT parameter (s)	Therapeutic effect	Consequences
A coumarin-based fluorescent probe for NIR imaging-guided photodynamic therapy against *S. aureus*-induced infection in mouse models	Coumarin	PDT light dose: 660 nm, NIR laser irradiation Excitation (λ_ex_): ~660 nm Absorption (λ_abs_): ~450 nm (Visible–NIR region) Absorption maximum (λ_abs max_): ~450 nm (Coumarin core) Emission (λ_em_): ~670 nm (NIR fluorescence) Singlet oxygen quantum yield (Φ_Δ_): high, sufficient for effective PDT	Antimicrobial	The probe generated reactive oxygen species under 660 nm irradiation, leading to efficient bacterial eradication, and its NIR emission enabled deep-tissue imaging, improving diagnostic and therapeutic precision with the high singlet oxygen yield to ensure potent PDT efficacy against S. aureus infections *in vivo*, demonstrating translational potential (Li et al., 2022)
Synthesis of coumarin-based photosensitizers for enhanced antibacterial type I/II photodynamic therapy		PDT light dose: 420–450 nm, Blue light irradiation Excitation (λ_ex_): ~420 nm Absorption (λ_abs_): ~410–430 nm Absorption maximum (λ_abs max_): ~420 nm Emission (λ_em_): ~460–550 nm (Visible fluorescence) Singlet oxygen quantum yield (Φ_Δ_): 0.839	Antibacterial	The coumarin-based PS generated singlet oxygen and free radicals, which enabled dual Type I/II PDT activity, and its high quantum yield ensured potent oxidative stress against *S. aureus*, even under challenging conditions (Ma et al., 2024)
Antifungal activity of coumarin against *Candida albicans* is related to apoptosis		PDT light dose: Not explicitly reported Excitation (λ_ex_): ~340–360 nm (UV–blue region) Absorption (λ_abs_): ~320–380 nm Absorption maximum (λ_abs max_): ~350 nm Emission (λ_em_): ~400–480 nm (Blue fluorescence) Singlet oxygen quantum yield (Φ_Δ_): <0.1	Antifungal	Coumarin induced apoptosis in *Candida albicans* by triggering mitochondrial Ca²^+^ influx and oxidative stress, and its singlet oxygen yield was relatively low, coumarin still disrupted fungal viability through ROS-mediated pathways (Jia et al., 2019)
Coumarin-tagged dinuclear trithiolato-bridged ruthenium(II)·arene complexes: Photophysical properties and antiparasitic activity		PDT light dose: Not explicitly reported Excitation (λ_ex_): ~340–360 nm (UV–blue region) Absorption (λ_abs_): ~320–380 nm (Broad UV–visible absorption) Absorption maximum (λ_abs max_): ~350 nm (Coumarin tag; quenched in Ru complexes) Emission (λ_em_): ~400–480 nm (Blue fluorescence) Singlet oxygen quantum yield (Φ_Δ_): Not reported	Antiparasitic	The coumarin–ruthenium complexes displayed potent antiparasitic activity against *Toxoplasma gondii*, which revealed mitochondrial damage, consistent with ROS-mediated stress typical of PDT (Desiatkina et al., 2020)
Curcumin-mediated antimicrobial photodynamic therapy prevents *Candida* biofilm formation in a species-dependent manner	Curcumin	PDT light dose: 450 nm, LED light irradiation Excitation (λ_ex_): 450 nm (Blue light) Absorption (λ_abs_): 400–500 nm Absorption maximum (λ_abs max_): ~430 nm (Curcumin’s main absorption band) Emission (λ_em_): 500–550 nm Singlet oxygen quantum yield (Φ_Δ_): Not reported	Antimicrobial	Curcumin-mediated PDT significantly inhibited *Candida* biofilm formation for the reductions of 86.8%, and the preventive effect was strong, but activity against mature biofilms was limited and highly species-dependent (da Silva et al., 2026)
aPDT for oral decontamination of hospitalized patients with COVID 19		PDT light dose: 450 nm, LED light irradiation, ~30–50 J/cm² Excitation (λ_ex_): 450 nm (Blue light) Absorption (λ_abs_): 400–500 nm Absorption maximum (λ_abs max_): ~430 nm Emission (λ_em_): 500–550 nm Singlet oxygen quantum yield (Φ_Δ_): Not directly reported in this study	Antiviral	Curcumin-mediated PDT reduced the oral microbial load and contributed to lowering SARS-CoV-2 presence in hospitalized COVID-19 patients, which was safe, species-dependent, and effective in preventing biofilm formation though standardized light doses and ROS yield measurements (Morimoto et al., 2022)
Antifungal effect of photodynamic therapy mediated by curcumin on *Candida albicans* biofilms *in vitro*		PDT light dose: 450 nm, LED light irradiation, ~30 J/cm² Excitation (λ_ex_): 450 nm Absorption (λ_abs_): 400–500 nm Absorption maximum (λ_abs max_): ~430 nm Emission (λ_em_): 500–550 nm Singlet oxygen quantum yield (Φ_Δ_): Not directly reported in this study	Antifungal	Curcumin-mediated PDT significantly reduced the viability of *Candida albicans* biofilms *in vitro*, and the antifungal effect was dose-dependent, with higher curcumin concentrations and light fluence producing stronger inhibition (Ma et al., 2019)
Evaluation of the photodynamic therapy with curcumin on *L. braziliensis* and *L. major* amastigotes		PDT light dose: 450 nm, LED light irradiation, ~30 J/cm² Excitation (λ_ex_): 450 nm Absorption (λ_abs_): 400–500 nm Absorption maximum (λ_abs max_): ~430 nm Emission (λ_em_): 500–550 nm Singlet oxygen quantum yield (Φ_Δ_): Not directly reported in this study	Antiparasitic	Curcumin-mediated PDT increased the polarity of mitochondrial membrane and a decrease in the number of parasites, *L. braziliensis* and *L. major* amastigotes to inactivate infected macrophages (Pereira et al., 2021)
Application of antimicrobial photodynamic therapy for inactivation of *acinetobacter baumannii* Biofilms	Riboflavin	PDT light dose: 450 nm, LED light irradiation, ~30 J/cm² Excitation (λ_ex_): 450 nm Absorption (λ_abs_): 350–480 nm Absorption maximum (λ_abs max_): ~445 nm Emission (λ_em_): 520 nm Singlet oxygen quantum yield (Φ_Δ_): ~0.5	Antimicrobial	Riboflavin-mediated PDT effectively disrupted *acinetobacter baumannii* biofilms, reducing bacterial viability and biofilm integrity (Buchovec et al., 2022)
Anti-viral photodynamic therapy in covid-19 management: A novel approach in treating patients in early infection stages		PDT light dose: 450 nm, LED light irradiation, ~30–50 J/cm² Excitation (λ_ex_): 445 nm Absorption (λ_abs_): 350–480 nm Absorption maximum (λ_abs max_): ~445 nm Emission (λ_em_): 520 nm Singlet oxygen quantum yield (Φ_Δ_): ~0.5	Antiviral	Riboflavin-mediated PDT was applied for prevention after contact with infected people or in case of positive testing but also in early cases with mild to moderate clinical symptoms (Weber et al., 2021)
*In Vitro* antifungal efficacy of blue-light photodynamic therapy with curcumin and riboflavin formulation activated by 450 nm diode laser against *Candida albicans* biofilm on titanium implants		PDT light dose: 450 nm, Diode laser irradiation, ~30 J/cm^2^ Excitation (λ_ex_): 450 nm Absorption (λ_abs_): 350–480 nm Absorption maximum (λ_abs max_): ~445 nm Emission (λ_em_): 520 nm Singlet oxygen quantum yield (Φ_Δ_): ~0.5	Antifungal	Riboflavin-mediated PDT effectively reduced *Candida albicans* biofilm viability on titanium implant surfaces in managing peri-implant fungal infections (Warakomska et al., 2025)
Complete inactivation of blood borne pathogen *Trypanosoma cruzi* in stored human platelet concentrates and plasma treated with 405 nm violet-blue light		PDT light dose: 405 nm, Violet-blue LED irradiation, ~75–100 J/cm² Excitation (λ_ex_): 405 nm Absorption (λ_abs_): 350–450 nm Absorption maximum (λ_abs max_): ~405 nm Emission (λ_em_): 520 nm Singlet oxygen quantum yield (Φ_Δ_): Not directly reported in this study	Antiparasite	Riboflavin-mediated PDT inactivated *Trypanosoma cruzi* in stored human platelet concentrates and plasma, as well as provided a proof of concept toward developing 405 nm light treatment as a pathogen reduction technology (PRT) (Jankowska et al., 2020)
Hypericin and pheophorbide a mediated photodynamic therapy fighting MRSA wound infections: A translational study from *in vitro* to *in vivo*	PPa	PDT light dose: *In vitro* 40 mW/cm² for 20 min (~48 J/cm²); *In vivo*: 500 mW for 60s (~30 J/wound) Excitation (λ_ex_): 670 nm Absorption (λ_abs_): 650–680 nm Absorption maximum (λ_abs max_): ~670 nm Emission (λ_em_): ~675 nm Singlet oxygen quantum yield (Φ_Δ_): ~0.64	Antimicrobial	Pheophorbide A-mediated PDT significantly reduced MRSA viability *in vitro* with >1 log10 CFU reduction and achieved potent wound infection control *in vivo* (Chan et al., 2021)
A comparison between the photodynamic action of hypocrellin A and pheophorbide a against bacteria and SARS-CoV-2 virus: A mini-review		PDT light dose: 650 nm, Red light irradiation, ~30-50 J/cm^2^ Excitation (λ_ex_): 670 nm Absorption (λ_abs_): 650–680 nm Absorption maximum (λ_abs max_): ~670 nm Emission (λ_em_): ~675–685 nm Singlet oxygen quantum yield (Φ_Δ_): ~0.6–0.7	Antiviral	Pheophorbide a-mediated PDT effectively targeted viruses, such as SARS-CoV-2, which generation of cytotoxic ROS, leading to the death of infected cells (Law, 2025)
The photoactivated antifungal activity and possible mode of action of sodium pheophorbide a on *Diaporthe mahothocarpus* causing leaf spot blight in *Camellia oleifera*		PDT light dose: 650 nm, Red light irradiation, ~30-50 J/cm^2^ Excitation (λ_ex_): 670 nm Absorption (λ_abs_): 650–680 nm Absorption maximum (λ_abs max_): ~670 nm Emission (λ_em_): ~675–685 nm Singlet oxygen quantum yield (Φ_Δ_): ~0.6–0.7	Antifungal	Sodium pheophorbide a-mediated PDT inhibited Diaporthe mahothocarpus, the causative agent of leaf spot blight in *Camellia oleifera*, which induced ROS-mediated oxidative stress and cell death to destroy the cell membrane and hyphal morphology, as well as ultimately inhibiting the mycelial growth (Shi et al., 2024)
Photodynamic control of human pathogenic parasites in aquatic ecosystems using chlorophyllin and pheophorbid as photodynamic substances		PDT light dose: 650 nm, Red light irradiation, ~30-50 J/cm^2^ Excitation (λ_ex_): 670 nm Absorption (λ_abs_): 650–680 nm Absorption maximum (λ_abs max_): ~670 nm Emission (λ_em_): ~675–685 nm Singlet oxygen quantum yield (Φ_Δ_): ~0.6–0.7	Antiparasite	Pheophorbide a-mediated PDT effectively inactivated human pathogenic protozoan parasites in aquatic ecosystems, which was able to kill mosquito larvae and other small animals within a few hours (Wohllebe et al., 2009)
Hypericin-mediated photodynamic antimicrobial effect on clinically isolated pathogens	Hypericin	PDT light dose: Visible light irradiation, ~20–40 J/cm² Excitation (λ_ex_): ~590 nm Absorption (λ_abs_): 540–600 nm Absorption maximum (λ_abs max_): ~598 nm Emission (λ_em_): ~651 nm Singlet oxygen quantum yield (Φ_Δ_): ~0.73	Antimicrobial	Hypericin-mediated PDT significantly induced killing of Gram-positive methicillin-sensitive and resistant *S. aureus* cells, but was not effective on Gram-negative *E. coli* cells (Yow et al., 2012)
Antiretroviral activities of hypericin and rose bengal: photodynamic effects on friend leukemia virus infection of mice		PDT light dose: Visible light irradiation, ~20–40 J/cm² Excitation (λ_ex_): ~590 nm Absorption (λ_abs_): 540–600 nm Absorption maximum (λ_abs max_): ~598 nm Emission (λ_em_): ~651 nm Singlet oxygen quantum yield (Φ_Δ_): ~0.73	Antiviral	Hypericin-mediated PDT significantly inhibited friend leukemia virus replication and reduced viral infectivity *in vivo*, at low doses, < 11 micrograms (Stevenson and Lenard, 1993)
Photoactivity of hypericin: from natural product to antifungal application		PDT light dose: Visible light irradiation, ~20–40 J/cm² Excitation (λ_ex_): ~590 nm Absorption (λ_abs_): 540–600 nm Absorption maximum (λ_abs max_): ~598 nm Emission (λ_em_): ~651 nm Singlet oxygen quantum yield (Φ_Δ_): ~0.73	Antifungal	Hypericin-mediated PDT possessed the ability to permeate the lipid membrane and accumulate in different cytoplasmic organelles of eukaryotic cells in the antifungal application (Galinari et al., 2023)
Development of a novel formulation with hypericin to treat cutaneous *leishmaniasis* based on photodynamic therapy in *in vitro* and *in vivo* Studies		PDT light dose: Red light irradiation, ~20–40 J/cm² Excitation (λ_ex_): ~590 nm Absorption (λ_abs_): 540–600 nm Absorption maximum (λ_abs max_): ~598 nm Emission (λ_em_): ~651 nm Singlet oxygen quantum yield (Φ_Δ_): ~0.73	Antiparasite	Hypericin-mediated PDT significantly reduced Leishmania (Viannia) panamensis viability *in vitro* and improved lesion healing in murine cutaneous leishmaniasis models against intracellular amastigotes (Montoya et al., 2015)

Coumarin, curcumin, riboflavin, PPa, and hypericin demonstrated pathogen-specific activity, ranging from *S. aureus* eradication and Candida biofilm inhibition to SARS-CoV-2 reduction and Leishmania measurement. These examples underscore the versatility of natural PSs, their adaptability to different light sources, and their translational potential from *in vitro* studies to clinical and *in vivo* models.

## Discussion

aPDT has emerged as a promising alternative to conventional antimicrobials in the management of pathogenic infections. The light-activated TCM-derived PSs generate ROS, which effectively disrupt microbial membranes, proteins, and nucleic acids. This multi-target oxidative stress bypasses conventional resistance mechanisms, leading aPDT valuable against multidrug-resistant pathogens and biofilm-associated infections (Demidova and Hamblin, 2004; Huang et al., 2012).

Based on the findings, the efficacy of aPDT from TCM-derived PSs is strongly influenced by the ROS generation pathways of Type I/II under different oxygen conditions. Coumarin and curcumin primarily exploit the Type I mechanism, producing free radicals such as O_2_^•−^ and •OH through electron transfer reactions. They are effective in hypoxic environments, where oxygen is limited but free radicals remain active (Yuan et al., 2022; Ma et al., 2024).

In contrast, riboflavin, hypericin, and PPa exhibit high singlet oxygen quantum yields, favoring the Type II mechanism under normoxic conditions. ^1^O_2_ rapidly oxidizes lipids, proteins, and nucleic acids, leading to irreversible microbial damage (Crocker et al., 2022; Chan et al., 2021). The balance between Type I/II pathways ensures the aPDT maintains efficacy across diverse infection sites. Type II dominates in oxygen-rich tissues, while Type I sustains activity in hypoxic niches. This dual adaptability underscores the therapeutic versatility of TCM-derived PSs in aPDT (Huang et al., 2012; Ding et al., 2011). The type of mechanism, oxygen dependence, antimicrobial spectrum, clinical strength, limitation, and translation potential of the TCM-derived PSs in aPDT are compared in **Table 5**. The comparison highlights that riboflavin offers nutritional safety but limited penetration, whereas PPa and hypericin provide superior photophysics but require delivery optimization.

**Table 5:** Comparative features of TCM-derived PSs in aPDT.

TCM-derived PS	Preferred mechanism	Oxygen dependence	Antimicrobial spectrum	Clinical strength	Limitation	Translation potential
Coumarin	Type I & II (dual)	Hypoxia (radical generation)	Bacteria, viruses, fungi, parasites	Broad oxidative stress, adaptable absorption	Poor aqueous solubility, Φ_Δ_ (< 0.1)	Useful for probe development
Curcumin	Mainly Type I	Hypoxia (strong)	Bacteria, resistant *Candida*, parasites	Antioxidant, PS dual role	Poor solubility, rapid degradation	Nanocarriers improve systemic use
Riboflavin	Primarily Type II	Normoxia	Biofilms, fungi, parasites, SARS-CoV-2	Safe, nutritional relevance	Limited penetration depth	Potential for blood sterilization, limit hypoxic efficacy
PPa	Type II	Normoxia	Fungi, parasites, SARS-CoV-2, MRSA	Strong tissue penetration	Hydrophobicity, aggregation reduce bioavailability	Clinical PDT if delivery optimized
Hypericin	Type II	Normoxia	MRSA, *Candida*, *Leishmania*, viruses	High ROS yield	Hydrophobicity, risk of phototoxicity	Controlled dosing

Type I PSs (coumarin, curcumin) are effective in hypoxia, and Type II PSs (riboflavin, PPa, hypericin) excel in normoxia, which has strengths (nutritional safety, tissue penetration, high ROS yield) and limitations (poor solubility, phototoxicity), guiding translational strategies.

An essential dimension of PDT efficacy lies in the optimization of light sources and irradiation parameters. The wavelength, fluence, and fluence rate directly influence tissue penetration, ROS yield, and microbial eradication. Blue–green light (400–550 nm) is highly effective for activating curcumin and riboflavin but is limited to superficial infections because of shallow or limited penetration. In contrast, red and near-infrared light (600–750 nm) penetrates deeper tissues, making it suitable for hypericin and PPa, which absorb strongly in the therapeutic window (Agostinis et al., 2011). Fluence (J/cm²) must be balanced, as insufficient exposure results in suboptimal ROS generation, and excessive irradiance risks photobleaching or photothermal damage. Recent evidence highlights the benefits of fractionated or pulsed light delivery, which improves oxygen replenishment and enhances microbial killing efficiency. A recent study demonstrated that tailoring fluence rates and pulse intervals significantly improved PDT outcomes against resistant bacterial strains, underscoring the importance of physical parameters alongside PS chemistry (Delarampour et al., 2026; Jernej et al., 2025). Thus, integrating optimized irradiation protocols into PDT design is critical for maximizing antimicrobial efficacy, minimizing off-target toxicity, and advancing clinical translation.

More importantly, curcumin and hypericin not only disrupt microbial growth directly but also produce singlet oxygen or free radicals upon irradiation, amplifying pathogen destruction. This dual functionality is the best reason for using TCM-derived PSs more than synthetic PSs, as the synthetic PSs rely solely on photophysical precision and lack the pharmacological effects of TCM theory (Wang et al., 2023).

TCM-derived PSs have the TCM theory that aligns with pharmacological activities or outcomes, which are described in section 2. For instance, the TCM principle of “invigorate blood circulation” and “remove blood stasis”, which boosts the body’s immune system and improves circulation. This process of TCM-derived PSs helps to prevent and treat microbes, viruses, fungi, and parasites by clearing clogged tissues and delivering immune cells to the infection. Similarly, “clear heat” parallels anti-inflammatory and immunomodulatory actions, such as suppression of NF-κB and MAPK signaling, reduction of pro-inflammatory cytokines, and stabilization of oxidative stress (Fu et al., 2024). These effects translate into broad antimicrobial, antiviral, antifungal, and antiparasitic activity.

The concepts of “invigorating blood circulation” and “dispelling blood stasis” can be translated into measurable physiological and cellular processes in TCM theory, which are well recognized in modern biomedical science. These terms correspond to vasodilation, eNOS activation, and improved microcirculation, which collectively enhance tissue perfusion and facilitate immune cell trafficking to sites of infection. Enhanced circulation not only improves oxygen delivery but supports neutrophil and macrophage migration, thereby strengthening pathogen clearance. This mechanistic interpretation is consistent with coumarin’s activity described in **Table 1**, where its circulation-enhancing effects are linked to modulation of NF-κB and Akt-mTOR signaling pathways, providing a direct bridge between ethnopharmacological terminology and immunological regulation (Mishra et al., 2020; Mustafa, 2025).

Similarly, the TCM principle of “clearing heat” aligns with anti-inflammatory activity, which involves suppression of NF-κB and MAPK signaling cascades, leading to reduced expression of pro-inflammatory cytokines such as TNF-α and IL-6. PSs like hypericin exert measurable immunomodulatory effects that relate to the “heat-clearing” description by attenuating inflammatory mediators. Indeed, hypericin’s role in reducing MRSA virulence through sarA/mecA downregulation from **Table 1** exemplifies that traditional terminology may be mapped onto molecular pathways of inflammation and microbial pathogenicity (Wang et al., 2019; Yang et al., 2024).

Another concept of “resolving toxicity” acts as redox regulation and oxidative stress balance. This refers to modulation of glutathione and trypanothione pathways, which maintain cellular homeostasis and enable ROS-mediated pathogen destruction. Hypericin’s antileishmanial activity, characterized by necrosis-like parasite death and altered trypanothione or glutathione levels in **Table 1**, provides an example of how “resolving toxicity” correlates to biochemical measures of oxidative stress (Singh et al., 2015; Sytar et al., 2016).

These explanations establish a scientific bridge between TCM theory and modern biomedical science, ensuring accessibility for training in Western medicine, and preserving the ethnopharmacological context of coumarin, curcumin, riboflavin, PPa, and hypericin. Thus, the ethnopharmacological principles of circulation, heat-clearing, and detoxification can be reframed as vascular modulation, cytokine suppression, and redox regulation, respectively, providing a scientifically coherent framework for understanding the therapeutic relevance of TCM-derived PSs in PDT.

The TCM-derived PSs not only exert direct antimicrobial effects through ROS generation, but can also influence host immune pathways. For example, coumarin and curcumin modulate NF-κB and Akt-mTOR signaling, thereby reducing pro-inflammatory cytokines and enhancing immune cell function (Mishra et al., 2020; Hussain et al., 2022). Riboflavin contributes to immune regulation by augmenting pathogen clearance and modulating interferon responses (Farah et al., 2022; Jeong et al., 2025). Hypericin demonstrates “heat-clearing” activity that relates to suppression of MRSA virulence factors and attenuation of inflammatory mediators (Wang et al., 2019; Yang et al., 2024).

In 2025, Özay reported that the synergistic paradigm of natural plant products plus PDT extends beyond pathogen eradication to include systemic immunomodulation (Özay et al., 2025). PDT-induced ROS not only damages microbial structures. It acts as secondary messengers to influence cytokine release, dendritic cell activation, and adaptive immune responses. This dual action bridges ethnopharmacology with modern clinical immunology, providing a comprehensive framework for understanding how TCM-derived PSs contribute to infection control and immune homeostasis.

However, TCM-derived PSs such as coumarin, curcumin, riboflavin, PPa, and hypericin have notable disadvantages, including solubility and bioavailability that limit clinical translation. Nanotechnology is a strategy to amplify these effects by improving solubility, bioavailability, and targeted delivery (Liu et al., 2024), using curcumin as an example for the antimicrobial, antiviral, and antifungal effects. The nanotechnology strategies such as sol-oil nano-curcumin, nanoemulsions, and dendrosomal formulations overcome these drawbacks by enhancing stability, penetration, and ROS yield. Evidence has shown that nano-curcumin improves aPDT against *Enterococcus faecalis* (Soliman et al., 2025), *Lactobacillus casei* (Ahrari et al., 2023), and *S. mutans* (Pourhajibagher et al., 2021). This has also demonstrated antiviral efficacy against HPV-16 (Bonfim et al., 2020) and antifungal activity against *Candida albicans* (Katiraee et al., 2016) (**Table 6**). The findings highlight nano-curcumin as a versatile advanced PS with broad-spectrum therapeutic potential (Ghanimatdan et al., 2024). Other TCM-derived PSs such as coumarin, riboflavin, PPa, and hypericin are also likely to benefit from similar nanotechnological strategies by analogy, including encapsulation in liposomes, polymer conjugates, or nanoparticle carriers. Coumarin solid lipid nanoparticles effectively treat *S. aureus* by enhancing solubility and bioavailability. Riboflavin–gold nanoparticle systems achieve bacterial inactivation through photooxidation and ROS generation. Silver-PPa nanoconjugates show potent antibacterial activity against *E. coli* via ROS and membrane disruption. *Hypericum perforatum* nanostructured lipid carriers exhibit antimicrobial and antiherpetic effects, targeting *E. coli*, *P. aeruginosa*, and HSV-1 to improve therapeutic efficacy and stability. These advanced PS formulations address the intrinsic limitations of natural PSs, such as poor solubility, instability, and limited bioavailability for enhancing ROS generation, tissue penetration, and targeting accumulation at infection sites.

**Table 6:** Advanced PS of curcumin–based PDT.

Topic	Nano-type	PDT parameter (s)	Target	Consequence
Nanocurcumin mediated photodynamic therapy as a novel approach for root canal disinfection: ex-*vivo* study	Nano-curcumin (sol-oil technique)	PDT light dose: optimized fluence for ex-vivo canal treatment Excitation (λ_ex_): ~450 nm (Blue light) Absorption (λ_abs_): broad absorption in the visible range Absorption maximum (λ_abs max_): ~420–430 nm Emission (λ_em_): ~500–550 nm (Green fluorescence) Singlet oxygen quantum yield (Φ_Δ_): moderate, enhanced by nano-formulation	Antimicrobial, improve solubility and bioavailability of curcumin, effective penetration into root canal biofilms (Enterococcus faecalis, mixed bacterial flora)	Nanocurcumin offered an effective PS for enhancing aPDT for management of endodontic infections (Soliman et al., 2025)
Antimicrobial photodynamic therapy against *Lactobacillus casei* using curcumin, nano-curcumin, or erythrosine with a dental LED curing device	Nano-curcumin	PDT light dose: optimized fluence for ex-vivo canal treatment Excitation (λ_ex_): ~450 nm (Blue light) Absorption (λ_abs_): broad absorption in the visible range Absorption maximum (λ_abs max_): ~420–430 nm Emission (λ_em_): ~500–550 nm (Green fluorescence) Singlet oxygen quantum yield (Φ_Δ_): moderate, enhanced by nano-formulation	Antimicrobial, improve solubility and bioavailability of curcumin, targeting *Lactobacillus casei* biofilms	Nano-curcumin enhanced superior antibacterial efficacy compared to free curcumin, demonstrating effectiveness in dental applications (Ahrari et al., 2023)
Nano-curcumin-containing pulp capping agent combined with antimicrobial photodynamic therapy (*in vitro* study)	Nano-curcumin	PDT light dose: optimized fluence with dental LED curing device Excitation (λ_ex_): ~450 nm (Blue light) Absorption (λ_abs_): broad absorption in the visible range Absorption maximum (λ_abs max_): ~420–430 nm Emission (λ_em_): ~500–550 nm (Green fluorescence) Singlet oxygen quantum yield (Φ_Δ_): moderate, enhanced by nano-formulation	Antimicrobial, improve solubility and bioavailability of curcumin; targeting *S. mutans* and other cariogenic/endodontic bacteria	Nano-curcumin pulp capping agent combined with PDT showed durable antibacterial activity, effective biofilm disruption, and potential for clinical dental applications (Pourhajibagher et al., 2021)
Antiviral activity of curcumin-nanoemulsion associated with photodynamic therapy in HPV-16-transduced vulvar cell lines	Curcumin-nanoemulsion	PDT light dose: optimized fluence for cell line irradiation Excitation (λ_ex_): ~430–450 nm (Blue light) Absorption (λ_abs_): broad absorption in the visible range Absorption maximum (λ_abs max_): ~420–430 nm Emission (λ_em_): ~500–550 nm (Green fluorescence) Singlet oxygen quantum yield (Φ_Δ_): moderate, enhanced by nanoemulsion formulation	Antiviral, improve solubility and bioavailability of curcumin targeting HPV-16 variants in vulvar epithelial cell lines	Curcumin-nanoemulsion PDT significantly reduced HPV-16 viral activity, associated with photoactivation (Bonfim et al., 2020)
Inhibitory effects of dendrosomal nanocurcumin on *Candida albicans* and systemic candidiasis in BALB/c mice	Dendrosomal nanocurcumin	PDT light dose: optimized fluence for fungal inactivation Excitation (λ_ex_): ~430–450 nm (Blue light) Absorption (λ_abs_): broad absorption in the visible range Absorption maximum (λ_abs max_): ~420–430 nm Emission (λ_em_): ~500–550 nm (Green fluorescence) Singlet oxygen quantum yield (Φ_Δ_): moderate, enhanced by dendrosomal formulation	Antifungal, improve solubility and bioavailability of curcumin targeting *Candida albicans* biofilms and systemic candidiasis	Dendrosomal nanocurcumin significantly inhibited *Candida albicans* growth and reduced systemic candidiasis in mice for the prevention of fungal infections (Katiraee et al., 2016)

The nano-curcumin formulations improve solubility, stability, ROS yield, and enhance antimicrobial, antiviral, and antifungal efficacy across dental, HPV, and Candida models, which demonstrates nanotechnology’s role in overcoming curcumin’s intrinsic limitations.

The therapeutic potential of TCM-derived PSs in PDT arises from the interplay between molecular structure, photophysical behavior, ROS generation, and formulation strategies. Structural scaffolds such as coumarin’s benzopyrone or PPa’s porphyrin core dictate absorption spectra and Φ_Δ_, which in turn determine whether Type I or Type II pathways dominate under hypoxic or normoxic conditions. These photophysical properties directly influence antimicrobial mechanisms, ranging from lipid peroxidation and protein oxidation to biofilm disruption. Nanotechnology further bridges molecular design with clinical translation, as liposomes, polymeric nanoparticles, and stimuli-responsive carriers improve solubility, bioavailability, and targeted delivery. Curcumin’s extended π-conjugation enhances visible-light absorption but requires nanocarrier stabilization, while hypericin’s polycyclic system confers high ROS yield yet demands careful dosing to avoid phototoxicity. Thus, clinical applicability depends on aligning chemical architecture, photophysical efficiency, and delivery systems to achieve safe, effective infection control (**Table 7**).

**Table 7:** Potential advanced PS of coumarin, riboflavin, PPa, and hypericin.

Topic	Nano-type	Target	Consequence
Coumarin-encapsulated solid lipid nanoparticles as an effective therapy against methicillin-resistant *staphylococcus aureus*	Coumarin-loaded solid lipid nanocarrier	Antimicrobial, improve solubility, stability, and bioavailability of coumarin targeting *S. aureus*	Coumarin-loaded solid lipid nanocarrier effectively treated *S. aureus* infections and overcame antibiotic resistance (Alqarni et al., 2022)
Riboflavin-mediated photooxidation of gold nanoparticles and its effect on the inactivation of bacteria	Riboflavin and pectin-coated gold nanoparticles	Antimicrobial, improve solubility, stability, and bioavailability of riboflavin targeting *S. aureus* and *P. aeruginosa*	Riboflavin and pectin-coated gold nanoparticles enhanced the antibacterial activity by photodynamic inactivation under adequate irradiation (Rivas Aiello et al., 2020)
Enhanced biosafety, anticancer and antibacterial photodynamic activities using silver-pyropheophorbide-a nanoconjugates	silver-pyropheophorbide-a nanoconjugates	Antimicrobial, improve solubility, stability, and bioavailability of pyropheophorbide-a targeting *E.coli*	Silver-pyropheophorbide-a nanoconjugates revealed strong antibacterial activity against *E.coli* following internal reactive oxygen species generation and membrane disintegration (Namulinda et al., 2024)
Antimicrobial and antiherpetic properties of nanoencapsulated *hypericum perforatum* extract	Nanostructured lipid carriers with *Hypericum perforatum* L.	Antimicrobial and antiviral, improve solubility, stability, and bioavailability of *hypericum perforatum* targeting *E. coli*, *P. aeruginosa*, and HSV-1	Nanostructured lipid carriers with *Hypericum perforatum* L. expressed activity against *E. coli*, *P. aeruginosa*, and HSV-1 extracellular virions, or viral adhesion (Sotirova et al., 2025).

The nanocarrier systems (solid lipid nanoparticles, gold nanoparticles, silver nanoconjugates, lipid carriers) enhance solubility, bioavailability, and ROS generation, broadening therapeutic applications against bacteria, fungi, and viruses.

TCM-derived PS protocol heterogeneity remains a major challenge. For example, employing diverse light sources, irradiation times, and dosimetry limits reproducibility. Variability in wavelength and fluence selection further complicates optimization, particularly when balancing tissue penetration with ROS yield. Oxygen dependence is another barrier, since hypoxic infection sites reduce Type II ^1^O_2_ generation, necessitating strategies to enhance Type I radical pathways. Formulation challenges, including hydrophobicity, aggregation, and poor bioavailability, restrict systemic delivery and highlight the requirement for nanocarrier systems. The standardized clinical protocols are still unknown, only confined to *in vitro* and animal models. Despite harmonized methodologies and multicenter clinical trials, translation into routine practice remains uncertain. Addressing these limitations via protocol standardization, advanced formulations, and rigorous clinical validation will be essential to realize the therapeutic potential of TCM-derived PSs in aPDT.

Up to the present, studies on TCM-derived advanced PSs for coumarin, riboflavin, PPa, and hypericin emphasize that cancer therapy is more than the application against pathogenic infections, and many remain comparatively underexplored. PDT has gained traction in oncology because of the established clinical protocols and clear translational pathways. In contrast, the application of PDT to pathogenic infections faces challenges such as diverse pathogen biology, biofilm complexity, and limited clinical trials. Thus, expanding PDT with TCM-based PSs to treat bacterial, viral, and fungal infections represents an important future direction, offering the potential to bridge traditional pharmacology with modern nanomedicine.

## Conclusions

PDT with TCM–based PSs represents a promising non-antibiotic strategy for combating pathogenic infections. Natural products such as coumarin, curcumin, riboflavin, PPa, and hypericin demonstrate intrinsic antimicrobial, antiviral, antifungal, and antiparasitic activities, which are enhanced by light-activated ROS generation. They have diverse chemical scaffolds and photophysical properties, which enable broad-spectrum efficacy, biofilm disruption, and targeted pathogen eradication, even against multidrug-resistant strains.

Preclinical evidence consistently supports these effects across bacterial, viral, and fungal models, and advances in nanotechnology have improved solubility, bioavailability, and site-specific accumulation. However, most data remain *in vitro* or animal-based, and clinical validation is limited. Current challenges include protocol heterogeneity, variability in irradiation parameters, oxygen dependence, formulation instability, and the absence of standardized clinical protocols.

Future research should prioritize harmonized methodologies, rigorous clinical trials, and translational strategies that align chemical architecture, photophysical efficiency, and delivery systems with practical clinical decision-making. PDT with TCM-derived PSs offers an innovative and integrative approach to pathogenic infection, bridging traditional pharmacology with modern nanomedicine.

## Future aspects

Future development of PDT with TCM-derived advanced PSs is expected to emphasize nanotechnology-enabled clinical translation. Liposomes, dendrosomes, polymeric nanoparticles, and nanoemulsions can improve solubility, bioavailability, and targeted delivery. Most studies have concentrated on oncology applications, for example, riboflavin photoactivation by upconversion nanoparticles for cancer treatment (Khaydukov et al., 2016), pyropheophorbide-a-loaded PEG-PLGA nanoparticles for tumor PDT (Liu et al., 2016), and hypericin tetraether liposome conjugates with antitumor and antiangiogenic activity (Plenagl et al., 2019). Advanced strategies include stimuli-responsive nanocarriers, co-delivery systems combining PSs with immunomodulators or conventional drugs. Curcumin-integrated smart nanocarriers for targeted delivery of 5-fluorouracil in cancer therapy (Kikani et al., 2026), and AI-guided light dosimetry. This predicts optimal PDT parameters by analyzing genetics, biomolecules, and microbiology, enabling personalization in PS selection, such as type, concentration, duration, and radiation source optimization, such as wavelength, exposure time, and frequency. The AI-driven personalization allows real-time monitoring, improves therapeutic outcomes, and reduces microbial resistance risks (Pourhajibagher et al., 2025). These approaches may be extended to PDT with TCM-derived PSs, bridging oncology advances with applications of pathogenic infection in the future.
