# Supplementary material for: Traditional Chinese medicine-derived natural photosensitizers for photodynamic therapy against pathogenic infections
Source: Front Cell Infect Microbiol. 2026 Oct 23;16:1932770. doi: 10.3389/fcimb.2026.1932770 (PMC13741180; doi:10.3389/fcimb.2026.1932770)
Supplement: Supplementary file 1 [file DataSheet1.docx]

**Narrative Review Checklist**

| **Section/Topic** | **Item No** | **Item** | **Reported on Section/Paragraph** |
| --- | --- | --- | --- |
| **TITLE** | | | |
| Title | 1 | Identify the report as a Narrative Review or Literature Review. | Title: Traditional Chinese medicine-derived natural photosensitizers for photodynamic therapy against pathogenic infections |
| **ABSTRACT** | | | |
| Structured summary | 2 | Provide a structured summary with the subsections as: objective, background, methods, conclusion. | Abstract: Objective (evaluate natural PSs in TCM for aPDT); Background (ROS generation, intrinsic antimicrobial activity, limitations, nanotechnology advances); Methods (databases: PubMed, ScienceDirect, Scopus, Web of Science, CNKI; 1995–2026; no language restriction; keywords; duplicates removed; inclusion/exclusion criteria applied); Results (52 studies synthesized; photophysical features, chemical scaffolds, Type I/II mechanisms, nanotechnology-enhanced PSs); Conclusion (promise of TCM-based advanced PSs, challenges in pharmacokinetics, safety, and clinical translation). |
| **INTRODUCTION** | | | |
| Rationale/background | 3 | Describe the rationale for the review in the context of what is already known. | Introduction: Conventional antimicrobials face limitations (resistance, toxicity, incomplete eradication); rationale for exploring antimicrobial PDT as a non-antibiotic strategy; bridging TCM natural PSs with modern nanotechnology. |
| Objectives | 4 | Specify the key question(s) identified for the review topic. | Explore TCM-based PSs (coumarin, curcumin, riboflavin, pheophorbide a, hypericin) in PDT; examine mechanisms (ROS generation, biofilm disruption, broad-spectrum efficacy, targeted action); assess translational potential and challenges. |
| **METHODS** | | | |
| Research selection | 5 | Specify the process for identifying the literature search (e.g., years considered, language, publication status, study design, and databases of coverage). | Methods: Databases searched (PubMed, ScienceDirect, Scopus, Web of Science, CNKI); time range 1995–2026; no language restriction; keywords listed; duplicates excluded; inclusion criteria (studies on TCM-based PSs in PDT against pathogenic infections); exclusion criteria (conference abstracts, insufficient methodological detail); final selection of 52 studies critically appraised and synthesized. |
| **DISCUSSION/SUMMARY** | | | |
| Narrative | 6 | Discuss:  1) research reviewed including fundamental or key findings,  2) limitations and/or quality of research reviewed,  and 3) need for future research. | Key findings: Antimicrobial, antiviral, antifungal, antiparasitic effects of TCM-derived PSs; photophysical features; Type I/II mechanisms; nanotechnology-enhanced PSs. Limitations: Poor solubility, bioavailability, phototoxicity, limited clinical trials. Future research: Nanocarriers, AI-guided dosimetry, translational validation, and clinical trial development. |
| Summary | 7 | Provide an overall interpretation of the narrative review in the context of clinical practice for health professionals, policy development and implementation, or future research. | Conclusion: TCM-based advanced PSs show promise as integrative anti-infective therapies with broad-spectrum efficacy and reduced systemic toxicity. Clinical translation requires optimization of pharmacokinetics, safety, and standardized protocols. Future directions include nanotechnology-enabled delivery, AI-guided personalization, and integration of natural products with modern photomedicine. |

**Table S1.** 52 selected studies of TCM-based PS in PDT against pathogenic infection.

|  | **Natural PS** | **Pathogen/Target** | **Study Type** | **Findings** | **Reference** |
| --- | --- | --- | --- | --- | --- |
| **1** | **Coumarin** | S. aureus | *In vitro* | Antimicrobial activity, SAR halogenation improve efficacy | Mishra, Pandey (2020) |
| 2 | **Coumarin** | HIV, Influenza, EV71, CVA16 (Coxsackievirus A16) | Review | Inhibit viral entry/replication via NF‑κB, Akt‑mTOR | Mishra, Pandey (2020) |
| 3 | **Coumarin** | Candida albicans | *In vivo* | Apoptosis via mitochondrial Ca²⁺ influx | Jia, Zhang (2019) |
| 4 | **Coumarin** | Parasites (Toxoplasma gondii) | *In vitro* | Mitochondrial disruption | Desiatkina, Păunescu (2020) |
| 5 | **Coumarin** | Parasites (Leishmania) | Review | Antiprotozoal activity | Tariq, Khan (2025) |
| 6 | **Coumarin** | S. aureus (NIR probe) | *In vivo* | Imaging-guided PDT efficacy | Li, Tu (2022) |
| 7 | **Coumarin** | S. aureus (synthetic derivatives) | *In vitro* | Enhance dual Type I/II PDT | Ma, Luo (2024) |
| 8 | **Coumarin** | Source plants (Angelica sinensis, Peucedanum praeruptorum) | Ethnopharmacology | Anti-inflammatory, circulation | Choi, Song (2022); Lin, Liu (2024) |
| 9 | **Coumarin** | Nanocarrier | *In vitro* | Solid lipid nanoparticles improve solubility, anti-MRSA | Alqarni, Foudah (2022) |
| **10** | **Curcumin** | S. aureus, E. coli; Zika, Influenza, HSV | Review | Suppress virulence factors, block viral replication | Hussain, Alam (2022) |
| 11 | **Curcumin** | Resistant Candida albicans | *In vitro* | Inhibit resistant strains | Hajifathali, Lesan (2023) |
| 12 | **Curcumin** | Aquaculture parasites | *In vivo* | Terminate reproduction of tomonts | Liu, Zhang (2017) |
| 13 | **Curcumin** | Candida biofilm | *In vitro* | Prevent biofilm formation | da Silva, da Mata (2026) |
| 14 | **Curcumin** | COVID-19 oral decontamination | Clinical | Reduce microbial load, SARS-CoV-2 presence | Morimoto, Rosin (2022) |
| 15 | **Curcumin** | Candida albicans biofilm | *In vitro* | Antifungal activity | Ma, Shi (2019) |
| 16 | **Curcumin** | Leishmania spp. | *In vitro* | Inactivates amastigotes | Pereira, Marcolino (2021) |
| 17 | **Curcumin** | S. aureus biofilm | *In vitro* | Antibacterial PDT | Yuan, Liu (2022) |
| 18 | **Curcumin** | Nano-curcumin (root canal) | *In vivo* | Enhanced antimicrobial PDT | Soliman, Sabra (2025) |
| 19 | **Curcumin** | Nano-curcumin (dental biofilm) | *In vivo* | Superior antibacterial efficacy | Ahrari, Mazhari (2023) |
| 20 | **Curcumin** | Nano-curcumin pulp capping | *In vitro* | Durable antibacterial activity | Pourhajibagher, Ranjbar (2021) |
| 21 | **Curcumin** | Nanoemulsion (HPV-16) | *In vitro* | Antiviral PDT | Bonfim, Monteleoni (2020) |
| 22 | **Curcumin** | Dendrosomal nanocurcumin | *In vivo* | Antifungal, systemic candidiasis | Katiraee, Ashrafai Helan (2016) |
| 23 | **Curcumin** | Nanoemulsion (echinococcosis) | *In vivo* | Antiparasitic efficacy | Ghanimatdan, Sadjjadi (2024) |
| 24 | **Curcumin** | Source plant (Curcuma longa) | Ethnopharmacology | Qi movement, pain relief | Sharifi-Rad, Rayess (2020); Wasim, Azmi (2025) |
| **25** | **Riboflavin** | Host immunity | Review | Immunomodulation, pathogen clearance | Farah, Chin (2022) |
| 26 | **Riboflavin** | A. baumannii biofilm | *In vitro* | Biofilm disruption | Buchovec, Vyčaitė (2022) |
| 27 | **Riboflavin** | SARS-CoV-2 | Clinical | RNA-targeted PDT | Weber, Mehran (2021); Jeong, Na (2025) |
| 28 | **Riboflavin** | Cryptococcus neoformans | *In vivo* | Reduce fungal burden | Huang, Ge (2025) |
| 29 | **Riboflavin** | Schistosoma mansoni | *In vitro* | Inhibit protease SmCB1 | Cogo, Pavani (2024) |
| 30 | **Riboflavin** | Candida albicans biofilm | *In vitro* | Antifungal PDT | Warakomska, Kępa (2025) |
| 31 | **Riboflavin** | Trypanosoma cruzi | *In vitro* | Pathogen reduction in blood | Jankowska, Nagarkatti (2020) |
| 32 | **Riboflavin** | Derivatives | *In vitro* | Pathogen inactivation | Crocker, Lee (2022) |
| 33 | **Riboflavin** | Source plant (Lycium chinense) | Ethnopharmacology | Qi replenishment | Tuan, Zhao (2014); da Silva-Araújo, Toscano (2025) |
| 34 | **Riboflavin** | Nanoparticle system | *In vitro* | Gold nanoparticle photooxidation | Rivas Aiello, Ghilini (2020) |
| **35** | **PPa** | MRSA wound infection | *In vitro* & *In vivo* | Antimicrobial PDT | Chan, Dharmaratne (2021) |
| 36 | **PPa** | SARS-CoV-2 | *In vitro* | Virucidal activity | Jimenez-Aleman, Castro (2021) |
| 37 | **PPa** | Camellia oleifera fungus | *In vitro* | ROS-mediated cell death | Shi, Yang (2024) |
| 38 | **PPa** | Trypanosoma cruzi, Leishmania | *In vitro* | Antiparasitic activity | Tayler, Boya (2019) |
| 39 | **PPa** | Aquatic parasites | *In vitro* | Parasite inactivation | Wohllebe, Richter (2009) |
| 40 | **PPa** | Source plant (Morinda citrifolia) | Ethnopharmacology | Damp-heat, toxic accumulation | Ratnoglik, Aoki (2014); Liu, Wang (2015) |
| 41 | **PPa** | Nanoconjugates | *In vitro* | Silver-PPa antibacterial | Namulinda, Song (2024) |
| **42** | **Hypericin** | MRSA | *In vitro* | Downregulate sarA/mecA | Wang, Li (2019) |
| 43 | **Hypericin** | S. aureus (clinical isolates) | *In vitro* | Antimicrobial PDT | Yow, Tang (2012) |
| 44 | **Hypericin** | Candida spp. | *In vitro* | Antifungal activity | Sytar, Švedienė (2016) |
| 45 | **Hypericin** | Leishmania spp. | *In vitro* | Necrosis-like parasite death | Singh, Sarma (2015) |
| 46 | **Hypericin** | Leishmania (murine model) | *In vivo* | Lesion healing | Montoya, Daza (2015) |
| 47 | **Hypericin** | SARS-CoV-2 | *In vitro* | Antiviral, RdRp/3CLpro inhibition | Souza, Martins (2025) |
| 48 | **Hypericin** | Friend leukemia virus | *In vivo* | Antiviral PDT | Stevenson, Lenard (1993) |
| 49 | **Hypericin** | Plant pathogen (Clavibacter michiganensis) | *In vitro* | Seed decontamination | Jernej, Gschwendtner (2025) |
| 50 | **Hypericin** | Antifungal application | Review | ROS-mediated antifungal | Galinari, Bianchi (2023) |
| 51 | **Hypericin** | Source plant (Hypericum perforatum) | Ethnopharmacology | Heat clearing, detoxification | Danova, Motyka (2022); Yang, Chen (2024) |
| 52 | **Hypericin** | Nanostructured lipid carriers | *In vitro* | Antimicrobial, antiherpetic | Sotirova, Ivanova (2025) |
